# Comparison of rituximab induction and maintenance regimens in anti-neutrophil cytoplasmic antibodies (ANCA)-associated vasculitis: PK/PD modelling of ANCA and gammaglobulin levels in real-world patients

**DOI:** 10.1016/j.ebiom.2025.105989

**Published:** 2025-11-03

**Authors:** Blaise Pasquiers, Benoit Blanchet, Xavier Puéchal, Xavier Declèves, Pascal Cohen, Claire Goulvestre, Marion Casadevall, Inès Benhabiles, Michel Vidal, David Ternant, Benjamin Terrier, Alicja Puszkiel

**Affiliations:** a Université Paris Cité, INSERM, Optimisation Thérapeutique en Neuropharmacologie OTEN U1144, 75006, Paris, France; b PhinC Development, 36 Rue Victor Basch, Massy, France; c Biologie du Médicament – Toxicologie, Cochin University Hospital, AP-HP, Paris, France; d Université Paris Cité, Faculty of Pharmacy, CNRS UMR8038, Inserm U1268, CARPEM, Paris, France; e Department of Internal Medicine, National Referral Center for Rare Systemic Autoimmune Diseases, Hôpital Cochin, Assistance Publique-Hôpitaux de Paris, Paris, France; f Université Paris Cité, F-75006, Paris, France; g Laboratory of Immunology, Cochin University Hospital, AP-HP, Paris, France; h Université de Tours, EA 4245 T2I, Tours, France; i Service de Pharmacologie Médicale, CHRU de Tours, Tours, France

**Keywords:** Rituximab, Anti-neutrophil cytoplasmic antibodies, Gammaglobulins, Population pharmacokinetics, PK/PD, Anti-neutrophil cytoplasmic antibodies-associated vasculitis

## Abstract

**Background:**

We aimed to provide pharmacokinetic-pharmacodynamic (PK/PD) rationale for selecting the optimal induction and maintenance dosing regimens in ANCA-associated vasculitis (AAV) using a population modelling approach based on PK, ANCA, and gammaglobulins data from a real-world cohort.

**Methods:**

A total of 121 patients with 296 plasma rituximab concentrations (99 and 197 in the induction and maintenance phases, respectively), 439 ANCA levels and 559 gammaglobulin levels were included in the analysis. Simulations of induction (375 mg/m^2^ weekly for 4 weeks and 1000 mg on day 0 and 14) and maintenance regimens (500 mg every 6 months [Q6M], 500 mg Q6M starting at month 4, 1000 mg Q4M, 500 mg Q4M) were performed in 1000 virtual patients. Dosing regimens were compared using a clinical utility score that equally weighted the percentage of patients in serological remission and at risk of hypogammaglobulinaemia (<6 g/L).

**Findings:**

The PK/PD model satisfactorily described the relationship between rituximab, gammaglobulins and ANCA concentrations over time. Both induction regimens resulted in a similar number of patients achieving serological remission and hypogammaglobulinaemia at 6 months. In the maintenance phase, increasing the dose (to 1000 mg) or the frequency of administration (Q4M versus Q6M) was associated with a higher number of patients with serological remission at month 24, but also with a higher risk of hypogammaglobulinaemia. In the maintenance phase, 500 mg Q6M (start at month 4 or 6), had a significantly higher utility score than 1000 mg Q4M.

**Interpretation:**

This PK/PD study can inform clinical decisions regarding the choice between different rituximab induction and maintenance regimens in patients with AAV in daily practice.

**Funding:**

This study received no funding.

**Research in context**

**Evidence before this study**

In the treatment of antineutrophil cytoplasmic antibody (ANCA)-associated vasculitis (AAV), rituximab can be given as a 4-dose (375 mg/m^2^ weekly for 4 weeks) or 2-dose (1000 mg on day 0 and 14) regimen for the induction of remission. The induction is followed by the maintenance phase beginning 4–6 months after the first induction dose and aiming at preventing disease relapse. Rituximab can be given in maintenance either at a dose of 500 mg every 6 months (Q6M) for 18 months or at 1000 mg every 4 months (Q4M) for 20 months. In the absence of direct comparative clinical trials, the optimal rituximab dosing regimen, as well as the duration of maintenance phase, need to be better defined in order to improve the risk-benefit ratio and quality of life for patients. ANCA targeting proteinase 3 (PR3) and myeloperoxidase (MPO) are implicated in the pathogenesis of AAV, and are a promising biomarker of rituximab efficacy. Hypogammaglobulinaemia, related to B-cell depletion induced by rituximab, is a common adverse event resulting in increased susceptibility to infection. Therefore, both ANCA and gammaglobulins appear to be interesting surrogate biomarkers for the efficacy and safety of rituximab and their longitudinal dynamics could be used to quantitatively compare induction and maintenance regimens.

**Added value of this study**

Using data collected in real-world patients with AAV, a pharmacokinetic-pharmacodynamic (PK/PD) model was developed linking plasma rituximab concentrations with ANCA and gammaglobulin levels during induction and maintenance phases. Simulations with the final model showed that the 2- and 4-dose induction regimens are equivalent in terms of efficacy (serological remission as assessed by serum ANCA levels <20 U/mL) and safety (serum gammaglobulins <6 g/L). In the maintenance phase, when equal weighting was applied to efficacy and safety endpoints, 500 mg Q6M (start at month 4 or 6) had a significantly higher utility score than 1000 mg Q4M, due to lower number of patients with hypogammaglobulinaemia.

**Implications of all the available evidence**

This study provides PK/PD and pharmacoeconomic rationale for selecting between different rituximab induction and maintenance regimens in patients with AAV in daily clinical practice. In addition, rituximab dose could be further optimised based on monitoring of plasma rituximab concentrations during the maintenance phase. These data may help inform patients and clinicians when deciding the risks and benefits of rituximab treatment.

## Introduction

Granulomatosis with polyangiitis (GPA) and microscopic polyangiitis (MPA) are antineutrophil cytoplasmic antibody (ANCA)-associated vasculitides (AAV) that affect small blood vessels and can lead to potentially organ- and life-threatening complications.^1^^,^^2^ The current treatment strategy to induce remission is the combination of glucocorticoids (GCs) and cyclophosphamide or rituximab.^3^^,^^4^ Rituximab has been approved by the Food and Drug Administration (FDA) for remission induction in new-onset and relapsed severe GPA and MPA as a four-infusion regimen (375 mg/m^2^ weekly for 4 weeks) in combination with GCs.^5^ More recently, an alternative induction regimen consisting of two infusions of 1000 mg given two weeks apart (D0-D14), approved for rheumatoid arthritis, has been proposed for AAV.^6^ A meta-analysis concluded that there was no significant difference in terms of efficacy and safety at 6 months between these two regimens.^7^ However, no direct comparative prospective studies have been conducted.

Once remission is achieved, maintenance therapy is aimed at preventing disease relapse^8^ and begins 4–6 months^9^^,^^10^ after the first induction dose. Rituximab can be given in maintenance either at a dose of 500 mg every 6 months (Q6M) for 18 months (MAINRITSAN regimen)^9^ or at 1000 mg every 4 months (Q4M) for 20 months (RITAZAREM protocol^10^). Both of these regimens have been shown to be superior to azathioprine in preventing disease relapse^9^, ^10^, ^11^ but have never been compared in a randomised clinical trial. The optimal rituximab dosing regimen and duration of induction and maintenance therapy need to be better defined at the individual patient level in order to improve the risk-benefit ratio and quality of life for patients while reducing treatment costs.

Simulations using models linking drug plasma exposure (pharmacokinetics, PK) to drug effects (pharmacodynamics, PD) are increasingly used to support drug dose selection based on the relevant efficacy and safety biomarkers. Recently, Jamois et al. used PK/PD approach to identify safe and effective dosing regimens of rituximab in paediatric patients with GPA/MPA which led to regulatory approval in this population.^12^ This approach could be used to quantitatively compare different dosing regimens of rituximab in adult patients with AAV for selection of the best regimens in clinical practice.

ANCA targeting proteinase 3 (PR3) and myeloperoxidase (MPO) are implicated in the pathogenesis of AAV, and patients who become ANCA-negative after induction therapy may have a lower risk of relapse.^13^ In addition, hypogammaglobulinaemia is a common rituximab-related adverse event resulting in increased susceptibility to infection^14^ and it was associated with increased rituximab plasma concentration in maintenance phase in 35 patients with AAV.^15^ Therefore, both ANCA and gammaglobulins appear to be interesting surrogate markers for the efficacy and safety of rituximab. Nevertheless, PK/PD relationship including these biomarkers in a real-world population have never been described before.

This study aimed to 1) develop a PK/PD model of rituximab including efficacy (MPO-ANCA and PR3-ANCA) and safety (serum gammaglobulins) PD biomarkers in real-world patients with AAV and 2) compare different induction and maintenance regimens using the final PK/PD model.

## Methods

### Patients and treatment

This retrospective analysis included patients with AAV treated with rituximab at the Cochin University Hospital (Paris, France) between January 2019 and January 2023. Eligibility criteria included age over 18 years and at least one measurable rituximab plasma concentration either in the induction or maintenance phase, or both. In patients who received rituximab for induction remission, the dosing regimen consisted of 375 mg/m^2^ weekly for 4 weeks or 1000 mg on days 0 and 14 (D0-D14). In the maintenance phase, rituximab was administered at 500 mg every 6 months starting 4–6 months after the induction phase and lasting 18–36 months. For patients with rituximab concentrations available only in maintenance phase, all previous doses of rituximab were informed in the modelling dataset.

### Ethics

The study was approved by the local Ethics Committee of Cochin University Hospital for retrospective analysis (CLEP N°: AAA-2023-09045) and was conducted in accordance with the Declaration of Helsinki. This retrospective analysis, based on blood samples collected for routine care, do not require signed informed consent in France, in accordance with CNIL's MR-003 methodology for non-interventional health research when individuals have been informed and have not objected.

### Data collection

Demographic, biological and clinical data at the time of first rituximab administration (either as induction or maintenance treatment) were retrospectively collected from medical records and included sex, age, body weight (BW), body surface area (BSA), aspartate aminotransferase (AST) and alanine aminotransferase (ALT), glomerular filtration rate (eGFR) estimated using CKD-EPI Creatinine Equation, serum albumin, and C-reactive protein (CRP) levels. Ethnicity data were not collected, in accordance with French regulations. MPO-ANCA, PR3-ANCA, and gammaglobulin levels were collected at baseline (maximum two weeks before induction start), in the induction phase (if available) and before each infusion in the maintenance phase. MPO-ANCA and PR3-ANCA were measured in serum by marketed ELISA kits (reference EA 1211-9601 G and EA 1201-9601-2 G, respectively, EUROIMMUN, Germany).

### Plasma rituximab concentrations

Plasma rituximab concentrations were retrospectively determined in plasma remaining after standard biochemistry tests performed as part of routine care. Samples were collected and stored as part of RADIPEM biobank (DC-2019-3677) at Cochin University Hospital in Paris, France. Samples were drawn at trough (before administration) and/or about 30 min after the end of a 1-h infusion during induction and maintenance phase. Blood samples were collected in heparinised tubes, the remaining plasma was stored at −20 °C until analysis. Rituximab concentrations were measured by a liquid chromatography coupled to tandem mass spectrometry detection method using the mAbXmise extraction kit (Promise Proteomics, France) with a calibration range of 2–100 mg/L.^16^ Plasma samples with concentrations above 100 mg/L were further diluted in drug-free plasma and re-assayed.

### Pharmacokinetic-pharmacodynamic analysis

#### Software

Rituximab concentrations, gammaglobulins, and ANCA data were analysed using non-linear mixed effects modelling (population approach) in Monolix® Suite 2023R1 (Lixoft, Antony, France). The Stochastic Approximation Expectation-Maximisation (SAEM) algorithm was used to estimate model parameters. Simulations were performed in Simulx®. Data plotting was performed in R version 4.2.2 coupled with RStudio version 2023.

#### Model development and validation

The PK/PD modelling analysis consisted of describing the effect of plasma rituximab concentrations on gammaglobulin and ANCA levels over time. Below lower limit of quantification (BLOQ) and above upper LOQ (ULOQ) data were included in the computation of the likelihood using left-censoring method (Supplemental Material S1). The details of the model development (structural and statistical models) are described in Supplementary Material S2.

One- and two-compartment models with a linear, non-linear or a time-varying clearance (CL) from the central compartment were tested to describe concentration–time data of rituximab. Time-dependency on CL was tested using a hyperbolic or sigmoidal function as previously proposed by Petitcollin et al.^17^ (Supplementary Material S2).

An indirect response model with zero-order production and first-order elimination in which rituximab inhibited synthesis of gammaglobulins was tested to describe gammaglobulin dynamics. Both linear and Imax functions were tested to describe rituximab effect on gammaglobulins. The details and model equations are presented in Supplementary Material S2.

**Fig. 1:**
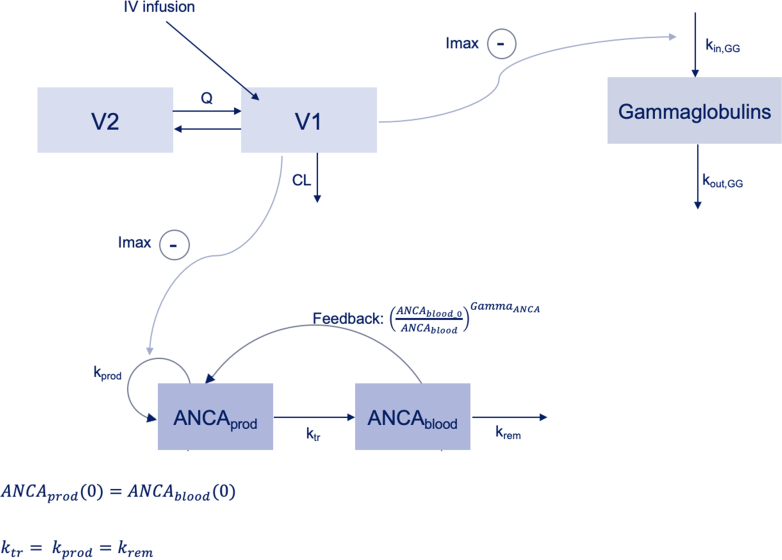
Schematic representation of the final pharmacokinetic-pharmacodynamic model. *CL*, rituximab clearance; *Q*, intercompartmental clearance; *V1*, central volume of distribution; *V2*, peripheral volume of distribution; *k*_*in,GG*_, gammaglobulin synthesis rate constant; *k*_*out,GG*_, gammaglobulin degradation rate constant. *ANCA*_*prod*_ is the amount of antineutrophil cytoplasmic antibodies (ANCA) in the deep (production) compartment, *ANCA*_*blood*_ is the amount of ANCA in the blood (circulating) compartment, *k*_*prod*_ is the first-order ANCA production rate constant, *k*_*tr*_ is the first-order transit rate constant and represents the inverse of mean transit time (MTT). *k*_*rem*_ is the first-order rate constant describing ANCA removal from the blood compartment. *Gamma*_*ANCA*_ is the power of negative feedback of the blood compartment on the deep compartment. Model equations are available in Supplementary Material S2.

Several structural models were tested including an indirect response model with a turnover of ANCA concentrations, a Friberg model^18^ which allows to consider oscillations in ANCA levels observed in some patients as well the structural model developed by Bensalem et al. based on data from the RAVE trial^19^ (Fig. 1, Supplementary Material S2). Both linear and Imax functions (with Imax fixed to 1) were tested to describe the inhibitory effect of rituximab on ANCA production. Because of sparse MPO-ANCA data, MPO- and PR3-ANCA were analysed together but separate parameters were tested and evaluated for model improvement using statistical criteria.

### Covariate analysis

The primary selection of covariates was based on biological and clinical relevance. The following covariates (at time of first rituximab infusion) were tested in the base models: age, sex, BSA, BW, CKD-EPI eGFR, serum albumin, CRP, CD19+, co-medications (prednisone, methotrexate). The details of the covariate analysis are presented in Supplementary Material S3.

### Model evaluation and validation

At each step, models were evaluated by visual inspection of goodness of fit (GOF) plots, stability and plausibility of parameter estimates, precision of estimations and statistical criteria (decrease in corrected Bayesian Information Criteria (BICc)). A non-parametric bootstrap (n = 200) was performed to assess model stability and precision of estimates. The final model was validated using a prediction-corrected visual predictive check (pcVPC) based on 500 simulations of the original dataset.

### Dose regimen simulations

The final PK/PD model was used for simulations of induction and maintenance regimens. The following regimens were evaluated for the induction phase: 1) 375 mg/m^2^ weekly for 4 weeks (Q1W); 2) 1000 mg D0-D14; and for the maintenance phase: 1) 500 mg every 6 months (Q6M, MAINRITSAN study protocol^9^) starting 6 months after the first induction dose until month 24; 2) 500 mg Q6M, starting 4 months after the first induction dose until month 22; 3) 500 mg every 4 months (Q4M) starting 4 months after the first induction dose until month 20; 4) 1000 mg Q4M (RITAZAREM study protocol^10^) starting 4 months after the first induction dose until month 20). In maintenance phase simulations, all virtual patients started induction phase with 1000 mg D0-D14 regimen.

For comparison of induction regimens, the percentage of patients in serological remission defined as MPO-ANCA or PR3-ANCA <20 U/mL, and with serum gammaglobulins <6 g/L (a cut-off associated with increased risk of infections^20^), and <4 g/L were extracted at 4 and 6 months. The median, 10th and 90th percentiles of plasma rituximab concentrations were calculated at the same time points.

The comparison of maintenance regimens was based on the percentage of patients in serological remission and with serum gammaglobulins <6 g/L and <4 g/L at month 12 and 24 after the first induction dose. These time points were selected based on the endpoint times in the MAINRITSAN2 and RITAZAREM clinical trials, and to ensure homogenous time points despite differences in the duration of the dosing regimens. The median, 10th and 90th percentiles of plasma rituximab steady-state trough concentration (C_trough,ss_) were extracted for each regimen. The percentage of patients with plasma rituximab concentration <4 mg/L at 3 months after the first maintenance dose was calculated for each regimen as it was previously reported to be independently associated with a higher risk of major relapse in patients with AAV.^21^ Details on the simulations are provided in Supplementary Material S4.

### Statistics

Simulated regimens were compared by calculating a clinical utility score that considers both the expected gain in efficacy and the increased risk of adverse events with increased drug exposure.^22^^,^^23^ Efficacy was characterised by achievement of serological remission and toxicity by hypogammaglobulinaemia (<6 g/L). Efficacy and safety were considered equally important in contributing to clinical utility (0.5/0.5). For each simulated dosing regimen (n = 1000 simulated patients each), the utility score was calculated using the following formula^23^:

$$Utility\;score=\omega_{eff}\;\cdot \;P_{eff}+\omega_{tox}\cdot \;\left(1-P_{tox}\right)$$

where ω_eff_ and ω_tox_ represent the contribution (weight) of efficacy and toxicity, respectively, to the utility score (fixed at 0.5 and 0.5, respectively). P_eff_ represents the proportion of patients achieving serological remission whereas P_tox_ represents the proportion of patients with hypogammaglobulinaemia (serum gammaglobulins <6 g/L). A supplemental analysis was performed, with hypogammaglobulinaemia defined as serum gammaglobulin levels <4 g/L. P_eff_ and P_tox_ were calculated at month 6 and 24 for induction and maintenance regimens, respectively. 90% prediction intervals were obtained by 100 replicates of the simulations of ANCA and gammaglobulins for each of the dosing regimen. In addition, a sensitivity analysis was conducted to test whether the assigned weight had a significant impact on utility score, with a weight allocation varying from 0.3/0.7 to 0.7/0.3 for ω_eff_/ω_tox_. The total cost of the different induction and maintenance regimens was calculated based on the price of rituximab (€1412 per 1 mg)^24^ and the daily care unit in France (€1900).

### Role of funders

There were no funders for this study.

## Results

### Patients and data

One hundred and twenty-one patients were enrolled. Demographic and biological characteristics of the study population at the time of the first rituximab administration are presented in Table 1. Among 102 patients who received rituximab in the induction phase, 70 (69%) received 375 mg/m^2^ Q1W regimen and 32 (31%) received 1000 mg D0-D14 regimen. For the remaining patients (n = 19), rituximab was administered in maintenance phase. A total of 296 plasma rituximab concentrations with a median number of 2 (range: 1–9) per patient were collected: 99 (33%), 197 (67%) during the induction and maintenance phase, respectively. Rituximab BLOQ concentrations accounted for 19% of the dataset (53 and 4 concentrations in the maintenance and induction phase, respectively). Trough concentrations represented 45% of the dataset (n = 133). The remaining concentrations included n = 125 taken less than 24 h after last infusion, 30 concentrations drawn around day 7, and 8 concentrations drawn around day 14 after last infusion (Figure S1). During data cleaning, less than 5% of the retrospectively collected concentration–time data were excluded due to inconsistencies between sampling time and observed rituximab concentration. Regarding PD data, 559 gammaglobulin levels were available (249 in the induction, 310 in the maintenance) and 439 ANCA measurements (215 in the induction, 224 in the maintenance), corresponding to 122 MPO-ANCA and 317 PR3-ANCA levels. There were 42 BLOQ and 13 ULOQ ANCA levels corresponding to 9.6% and 2.9% of the total dataset, respectively. ANCA levels were available at a median [5–95th percentile] time after last rituximab infusion of 5.3 [0–6.5] months. Gammaglobulins were available at a median time of 5.3 [0–6.4] months after last rituximab infusion. Three patients were excluded from ANCA modelling as they were identified as outliers based on high individual weighted residuals (IWRES >4). Plots representing raw rituximab, ANCA and gammaglobulins concentration–time data are presented in Figures S1–S3.

**Table 1:** Patients’ characteristics at time of first rituximab administration (n = 121).

Characteristic	Median [IQR] or number (%)
Age (years)	59 [40–72]
Female^a^	66 (55)
Body weight (kg)	69 [58–75]
BSA (m^2^)	1.78 [1.63–1.91]
Type of vasculitis	
GPA	94 (78)
PAM	20 (16)
EGPA	1 (1)
Cryoglobulinaemic vasculitis^b^	6 (5)
ANCA status^c^	
PR3	67 (58)
MPO	35 (31)
Negative	13 (11)
Organ involvement at diagnosis	
Myalgia	24 (20)
Arthralgia	46 (38)
Fever	19 (16)
Weight loss	35 (29)
Skin	25 (21)
Eye	25 (21)
ENT	64 (53)
Chest	51 (42)
Cardiovascular	7 (6)
Abdominal	3 (2)
Kidney	46 (38)
Nervous system	28 (23)
CD19+ (cells/μL)	49 [18–170]
eGFR (mL/min/1.73 m^2^)^d^	88 [63–105]
CRP (mg/L)	1.8 [0.5–4.8]
Serum albumin (g/L)	41 [38–44]

BSA, Body Surface Area; CRP, C-reactive protein; eGFR, estimated glomerular filtration rate; EGPA, eosinophilic granulomatosis with polyangiitis; GPA, granulomatosis with polyangiitis; ENT, ear, nose and throat; MPO-ANCA, antineutrophil cytoplasmic antibodies targeting myeloperoxidase; PAM, microscopic polyangiitis; PR3-ANCA, antineutrophil cytoplasmic antibodies targeting proteinase 3.

a Sex was self-reported by study participants.

b Patients with cryoglobulinaemic vasculitis were included only in the rituximab pharmacokinetic model.

c Among 115 patients with ANCA-associated vasculitis.

d eGFR was estimated using CKD-EPI Creatinine Equation.

### PK/PD modelling analysis

**Fig. 2:**
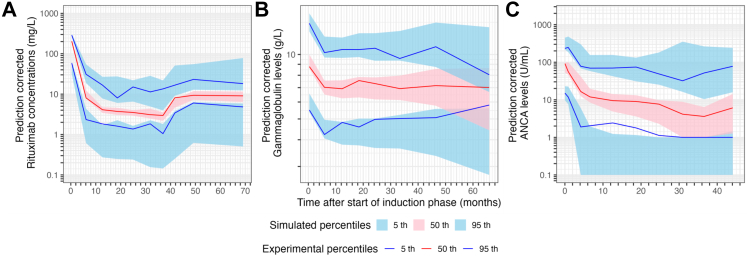
Prediction-corrected visual predictive checks (n = 500 simulations of the original dataset) of the final models for A) rituximab, B) gammaglobulins, and C) ANCA levels.

Rituximab concentration–time data were described using a two-compartment model (decrease of 187 points in BICc compared to a one compartment model) parametrised in terms of central (V1) and peripheral (V2) volumes of distribution, intercompartmental clearance (Q) and linear (non-saturable) elimination clearance (CL) (Fig. 1). Inclusion of non-linear or time-varying CL was associated with high imprecision in parameter estimation. Inter-individual variability (IIV) was included on CL and V1 and could not be reliably estimated on V2 and Q, and was therefore fixed to 0 (Table 2). Proportional model for residual unexplained variability was associated with the lowest BICc. GOF (Figure S4) and pcVPC of the final model (Fig. 2, Figure S7) did not show major model misspecifications. The mean estimates from the original model were included in the 95% confidence intervals obtained by 200 bootstrap analyses (Table 2), confirming model stability. The only parameter with an important difference (50%) between the mean estimate and median obtained by bootstrap was Q which could be explained by sparse sampling in the distribution phase.

**Table 2:** Mean parameter estimates of the final PK/PD model and results of the non-parametric bootstrap analysis.

Parameter	Mean estimate (RSE%) [shrinkage]	Non-parametric bootstrap^a^ median [CI 95%]
**Fixed effect**s		
CL (L/day)	0.15 (3.4)	0.15 [0.13–0.18]
Effect of age on CL	−0.33 (23.7)	−0.23 [-0.43; −0.068]
Effect of sex on CL	−0.23 (25.7)	−0.35 [-0.60; −0.11]
V1 (L)	2.61 (2.4)	2.54 [2.20–2.73]
Effect of BW on V1	0.71 (15.2)	0.75 [0.50–1.07]
V2 (L)	3.01 (3.1)	3.11 [2.40–3.87]
Q (L/day)	0.52 (1.9)	1.04 [0.34–3.42]
BASE_GG_ (g/L)	9.35 (3.0)	9.35 [8.76–10]
k_out,GG_ (day^−1^)	0.0034 (7.0)	0.0035 [0.0022–0.005]
IC_50,GG_ (mg/L)	41.8 (7.5)	40.0 [25.8–65.0]
BASE_ANCA_ (U/mL)	75.6 (12.0)	76.6 [59.7–101]
Effect of CRP on BASE_ANCA_	0.21 (28.3)	0.19 [0.07–0.32]
MTT_ANCA_ (days)	31.9 (13.7)	29.2 [21.4–38.9]
Gamma_ANCA_	0.03 (25.2)	0.027 [0.012–0.038]
IC_50,ANCA_ (mg/L)	264 (29.4)	296 [184–588]
Effect of PR3-ANCA on IC_50,ANCA_	−0.96 (25.1)	−0.94 [-1.53; −0.40]
**Random effects**		
ω_CL_	0.38 (10.6) [12]	0.38 [0.30–0.47]
ω_V1_	0.15 (15.5) [42]	0.16 [0.07–0.24]
ω_BASE,__G__G_	0.29 (7.4) [5.8]	0.29 [0.23–0.33]
ω_BASE,ANCA_	1.06 (8.7) [8.7]	1.11 [0.85–1.34]
ω_MTT,ANCA_	0.66 (11.7) [25]	0.65 [0.41–0.86]
ω_IC50,ANCA_	0.80 (13.3) [28]	0.75 [0.48–1.01]
**Residual error model**		
Rituximab σ_prop_	0.21 (3.4)	0.21 [0.16–0.25]
Gammaglobulins σ_prop_	0.17 (3.5)	0.17 [0.15–0.19]
ANCA σ_prop_	0.40 (3.7)	0.40 [0.33–0.45]

ANCA, antineutrophil cytoplasmic antibodies; BASE, baseline; BW, body weight; CL, clearance; CI95%, confidence interval at 95%; CRP, C-reactive protein; GG, gammaglobulins; IC_50_, half-maximal inhibitory concentration; k_out_ first-order degradation rate constant; MPO-ANCA, antineutrophil cytoplasmic antibodies targeting myeloperoxidase; MTT, mean transit time; PR3-ANCA, antineutrophil cytoplasmic antibodies targeting proteinase 3; RSE, relative standard error; Q, intercompartmental clearance; V1, central volume of distribution; V2, peripheral volume of distribution.

a Medians and CI 95% were obtained with 200 non-parametric bootstrap runs.

An indirect response model was used to describe the concentration–time data of gammaglobulins in which rituximab inhibited gammaglobulin zero-order synthesis rate constant (k_in,GG_) with a half-maximal inhibitory concentration (IC_50,GG_) of 41.8 mg/L (relative standard error [RSE] = 7.5%) (Fig. 1). The model provided a good description of the data as shown by proportional residual error of 17% (Table 2), GOF (Figure S5) and pcVPC (Fig. 2) without any significant model misspecifications.

Individual parameter estimates from the model including rituximab and gammaglobulins were used to generate individual PK and PD profiles as an input to the ANCA PK/PD model (sequential modelling). The final PK/PD model for ANCA consisted of the structural model reported by Bensalem et al. based on data from the RAVE trial^19^ (Fig. 1). The variations of this model with one or two transit compartments were associated with high imprecision in parameter estimates (RSE >50%). The model parameters included baseline ANCA levels (BASE_ANCA_), mean transit time (MTT_ANCA_) which covers mean time of ANCA production (k_prod_), ANCA move from deep to circulating compartment (k_tr_), and ANCA degradation (k_rem_), Gamma_ANCA_ which represents the magnitude of the negative feedback of circulating ANCA on the formation of ANCA and concentration of rituximab producing 50% maximal inhibition of ANCA production (IC_50,ANCA_). The attempts to analyse separately MPO-ANCA and PR3-ANCA resulted in model instability, probably because of a low number of MPO-ANCA titres and sparse sampling. Therefore, only rituximab half-maximal inhibitory concentration was estimated separately for MPO-ANCA and PR3-ANCA (mean IC_50,ANCA_ of 264 mg/L and 101 mg/L, respectively, Wald test p-value <0.0001). Mean estimated MTT was 31.9 days for both MPO-ANCA and PR3-ANCA. GOF are presented in Figure S6, and pcVPC of the final model in Fig. 2 and Figure S8 (stratified on MPO- and PR3-ANCA). Although a slight underprediction of the 5th percentile was observed, the predictive performance of the final model was deemed satisfactorily for simulations, as the cut-off value used in simulations to distinguish between negative or positive ANCA levels (20 U/mL) lies above the 5th percentile.

### Covariate analysis

Among the tested covariates, sex and age had a significant impact on CL (Wald test p-value = 0.0001 and < 0.0001, respectively) as well as BW on V1 (p-value <0.0001). CL was 20% lower in female compared to male patients and 7.3% lower in a 72-year old patient (95th percentile) compared to a typical 58-year old patient (weighted mean). V1 was 24% higher in a patient with BW of 93 kg (95th percentile) compared to a patient with BW of 69 kg (weighted mean).

CRP was correlated with BASE_ANCA_ (Wald test p-value = 0.0004) with a 35% higher BASE_ANCA_ in a patient with CRP of 45 mg/L (3rd quartile in the studied population) compared to a patient with a CRP of 11 mg/L (weighted mean). The equations of the final covariate model are presented in Supplementary Material S5.

### Dose regimen simulations

#### Induction phase

**Fig. 3:**
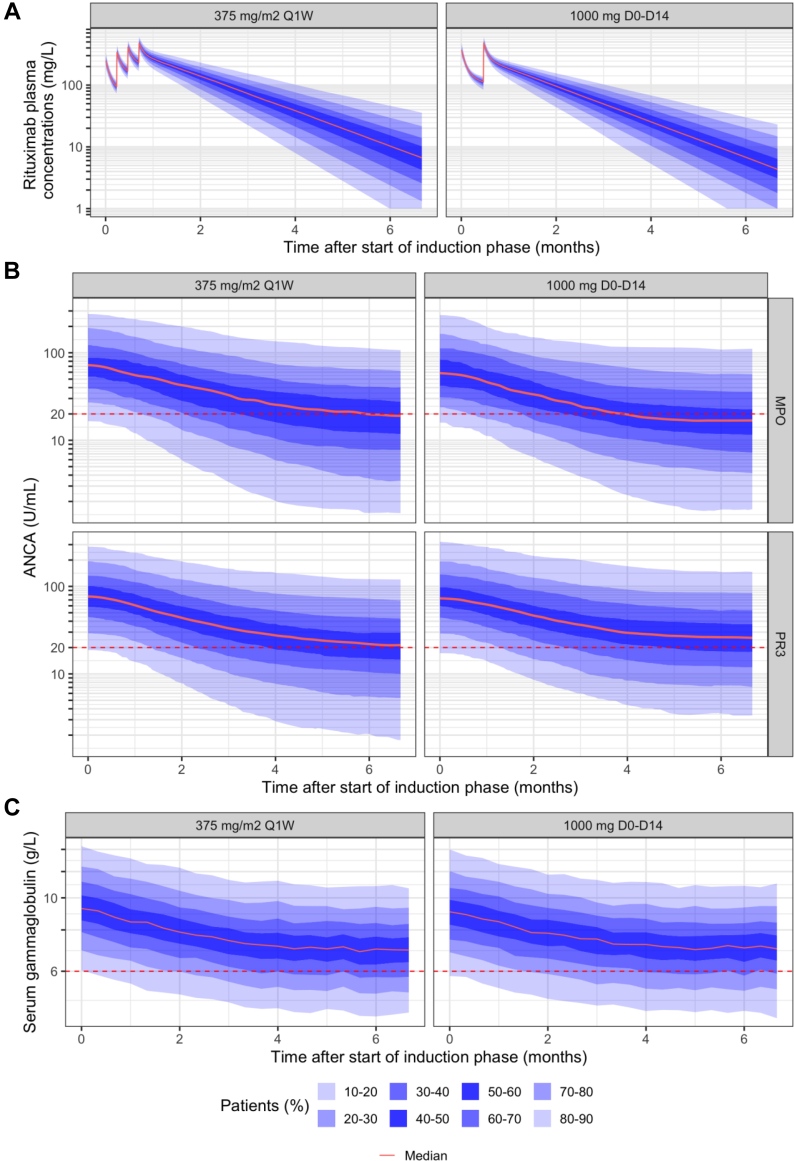
Simulations (n = 1000 patients) of induction regimens: A) rituximab plasma concentrations; B) MPO-ANCA and PR3-ANCA levels; C) serum gammaglobulin levels versus time after start of induction phase. Red dashed lines represent the cutoff value of 20 U/mL for ANCA and 6 g/L for serum gammaglobulins. ANCA, antineutrophil cytoplasmic antibodies; MPO, Myeloperoxidase; PR3, Proteinase 3.

The two induction regimens showed similar PK, gammaglobulin and ANCA profiles over time (Fig. 3), resulting in a similar number of patients achieving serological remission at 4 and 6 months after the first induction dose. The percentage of patients achieving negative MPO-ANCA at 6 months was 50% and 55% with 375 mg/m^2^ Q1W and 1000 mg D0-D14, respectively. For PR3-ANCA, these percentages were 46% and 42% for 375 mg/m^2^ Q1W and 1000 mg D0-D14, respectively (Table 3). Simulated gammaglobulin profiles were comparable between the two regimens with 34% of patients presenting gammaglobulins <6 g/L at month 6. Table S1 presents the percent decrease from baseline in ANCA levels at 6 months.

**Table 3:** Comparison of rituximab induction regimens using simulations with the final pharmacokinetic-pharmacodynamic model.

	375 mg/m^2^ Q1W	1000 mg D0-D14
**Median plasma rituximab concentration (mg/L) (10th–90th percentile)**		
4 Months	38.5 (7.94, 101)	25.4 (5.32, 67.9)
6 Months	10.3 (<1.0, 46.1)	6.78 (<1.0, 30.0)
**MPO-ANCA < 20 U/mL**		
4 Months	45%	50%
6 Months	50%	55%
**PR3-ANCA < 20 U/mL**		
4 Months	41%	38%
6 Months	46%	42%
**Gammaglobulins < 6 g/L**		
4 Months	30%	31%
6 Months	34%	34%
**Gammaglobulins < 4 g/L**		
4 Months	4.6%	5.1%
6 Months	5.0%	5.1%

MPO-ANCA, antineutrophil cytoplasmic antibodies targeting myeloperoxidase; PR3-ANCA, antineutrophil cytoplasmic antibodies targeting proteinase 3; D0, day 0; D14, day 14; Q1W, every week.

Data are expressed as percentage of simulated patients (n = 1000 for each dosing regimen) or median value (10th, 90th percentile).

#### Maintenance phase

**Fig. 4:**
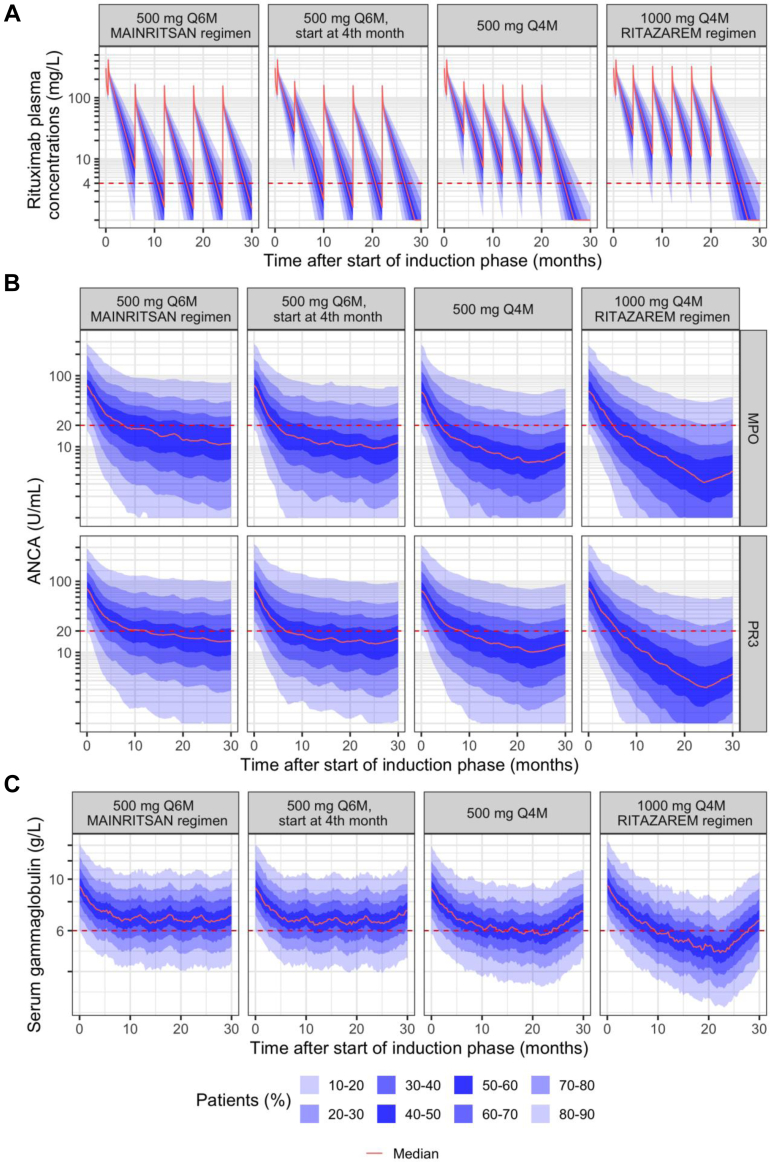
Simulations (n = 1000 patients) of maintenance regimens following induction phase with 1000 mg D0-D14: A) rituximab plasma concentrations; B) MPO-ANCA and PR3-ANCA levels; C) serum gamma globulin levels versus time after start of induction phase. Red dashed lines represent the cutoff values of 4 mg/L (rituximab), 20 U/mL (ANCA) and 6 g/L (gammaglobulins). ANCA, antineutrophil cytoplasmic antibodies; MPO, Myeloperoxidase; PR3, Proteinase 3.

Simulations of different maintenance regimens after an induction phase of 1000 mg on D0-D14 are shown in Fig. 4. The 1000 mg Q4M regimen (RITAZAREM) resulted in a higher number of patients with sustained serological remission compared to the 500 mg Q6M regimen (MAINRITSAN) (Table 4). At month 24, the percentage of patients with negative MPO-ANCA was 79% versus 59% for the RITAZAREM and MAINRITSAN regimens, respectively, while the percentage of patients with negative PR3-ANCA was 77% versus 46%. The regimen with lower dose (500 mg Q4M) maintained negative MPO-ANCA and PR3-ANCA in 75% and 65% of simulated patients, respectively, showing similar efficacy as the 1000 mg dose. Starting the maintenance phase earlier, i.e. 500 mg Q6M from month 4 versus 500 mg Q6M from month 6, was associated with a higher number of patients with negative MPO-ANCA and PR3-ANCA at month 24 (68% versus 59% for MPO-ANCA and 58% versus 46% for PR3-ANCA, respectively).

**Table 4:** Comparison of rituximab maintenance regimens using simulations with the final pharmacokinetic-pharmacodynamic model.

	500 mg Q6M (MAINRITSAN regimen)	500 mg Q6M, start at month 4	500 mg Q4W	1000 mg Q4W (RITAZAREM regimen)
**Rituximab C_trough,ss_ (mg/L)**^a^	1.53 (<1.0, 8.16)	1.61 (<1.0, 8.54)	5.77 (1.03, 19.2)	11.1 (1.81, 40.3)
**Rituximab concentration < 4 mg/L at month 3 after the 1st maintenance dose**	16%	13%	14%	6.2%
**MPO-ANCA < 20 U/mL**				
12 Months	52%	62%	67%	67%
24 Months	59%	68%	75%	79%
**PR3-ANCA < 20 U/mL**				
12 Months	49%	55%	56%	65%
24 Months	46%	58%	65%	77%
**Gammaglobulins < 6 g/L**				
12 Months	34%	43%	45%	52%
24 Months	35%	40%	47%	66%
**Gammaglobulins < 4 g/L**				
12 Months	6.3%	8.9%	12%	17%
24 Months	7.1%	10%	15%	29%

MPO-ANCA, antineutrophil cytoplasmic antibodies targeting myeloperoxidase; PR3-ANCA, antineutrophil cytoplasmic antibodies targeting proteinase 3; Q4W, every 4 months; Q6W, every 6 months.

Data are expressed as percentage of simulated patients (n = 1000 for each dosing regimen) or median value (10th, 90th percentile).

a C_trough,ss_ was extracted at month 20 for 500 mg Q4M and 1000 Q4M regimens, at month 22 for 500 mg Q6M start at 4 month regimen and at month 24 for 500 mg Q6M regimen.

In terms of safety, increasing dose and/or dosing frequency was associated with a higher number of patients with hypogammaglobulinaemia (Table 4). The highest proportion of patients with serum gammaglobulins <6 g/L was observed with the 1000 mg Q4M regimen (66% at 24 months) compared to the 500 mg Q6M regimen (35%).

### Clinical utility scores and treatment costs

**Fig. 5:**
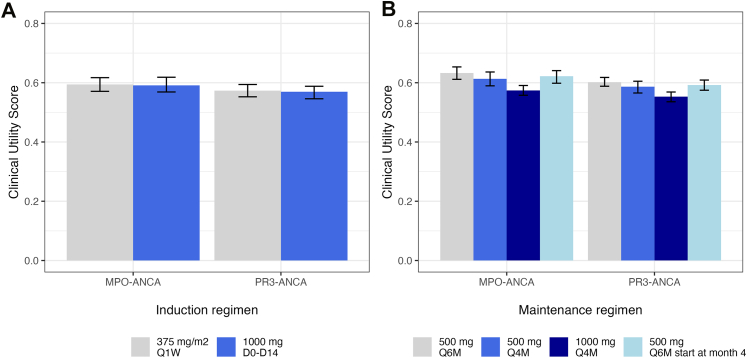
Clinical utility score for A) induction and B) maintenance regimens of rituximab based on simulations with the final model (n = 1000 patients for each regimen). Data are represented as median and 90% prediction interval obtained by 100 replicates of the simulations. In the calculation of utility score, equal weighting for efficacy (proportion of patients with negative ANCA titre) and safety (number of patients with hypogammaglobulinaemia, i.e. serum gammaglobulins <6 g/L) was applied (0.5/0.5). Proportion of patients with negative ANCA titre and hypogammaglobulinaemia was calculated at 6 and 24 months after first induction dose for induction and maintenance regimens, respectively. MPO-ANCA, antineutrophil cytoplasmic antibodies targeting myeloperoxidase; PR3-ANCA, antineutrophil cytoplasmic antibodies targeting proteinase 3.

Both induction regimens showed similar utility score with overlapping 90% prediction intervals (Fig. 5A). When serum gammaglobulins <4 g/L were considered, the utility score remained comparable (Figure S9). The sensitivity analysis, with varying efficacy/safety weight assignments, did not alter the conclusion that the two regimens are comparable (Figures S10 and S11). As 1000 mg D0-D14 regimen is associated with less visits to clinic, it has a lower treatment cost compared to 375 mg/m^2^ Q1W (€6624 versus €11,264, respectively, Table S2). Concerning maintenance regimens, 1000 mg Q4M regimen had significantly lower utility score than 500 mg Q6M (start at month 4 or 6), and a tendency was observed for 500 mg Q4M (Fig. 5B). This is due to a higher number of patients with hypogammaglobulinaemia with 1000 mg Q4M. When serum gammaglobulins <4 g/L were considered as the safety component, all maintenance regimens had comparable utility scores (Figure S12). Indeed, the utility score of the regimen with the least favourable safety profile increases more markedly than that of regimens in which the safety criterion contributes less to the calculation of the utility score. 500 mg Q4M yields a similar utility score than 1000 mg Q4M, while it has a lower treatment cost (€13,030 versus €16,560 per patient, respectively). The lowest treatment costs are associated with 500 mg Q6M regimens (€10,424, Table S2). The sensitivity analysis showed that when greater weight is assigned to safety over efficacy (0.3/0.7 for ω_eff_/ω_tox_), regimens with more frequent administration (500 mg Q4M and 1000 mg Q4M) yield lower utility score than 500 mg Q6M (Figure S13). However, when greater weight is assigned to efficacy over safety (0.7/0.3 for ω_eff_/ω_tox_), no statistically significant difference between regimens is observed for MPO-ANCA but 1000 mg Q4M yields a significantly higher utility score for PR3-ANCA than 500 mg Q6M regimen (Figure S14). This information may help to inform patients and clinicians when weighting the risks and benefits of rituximab treatment on an individual level.

## Discussion

This study describes PK/PD relationship between rituximab plasma concentrations, gammaglobulins and ANCA levels in an unselected real-world population with AAV using population approach. The simulations from the final PK/PD model showed that the two induction regimens (375 mg/m^2^ Q1W or 1000 mg D0-D14) are similar in terms of serological remission and hypogammaglobulinaemia at 6 months. In the maintenance phase, 1000 mg Q4M regimen had significantly lower utility score than 500 mg Q6M (start at month 4 or 6), due to a higher number of patients with hypogammaglobulinaemia. However, when greater weight is assigned to efficacy over safety, 1000 mg Q4M yields a significantly higher utility score for PR3-ANCA than 500 mg Q6M regimen.

Concentration–time data of rituximab were accurately described using a two-compartment model with linear elimination. Numerous population PK studies of rituximab in various diseases were reported in the literature with varying estimates of CL and distribution parameters.^25^, ^26^, ^27^, ^28^, ^29^, ^30^, ^31^, ^32^, ^33^ It has been shown that disease has a significant impact on rituximab elimination CL and volumes of distribution^33^^,^^34^ which might explain in part these differences. In our model, the mean estimate of elimination CL was 0.15 L/day, and the mean elimination half-life of rituximab derived from the model was 28 days, which is coherent with values reported in patients with AAV.^19^^,^^33^ In agreement with the literature, elimination CL was lower in older patients and in females whereas central volume of distribution (V1) increased with BW.^25^, ^26^, ^27^, ^28^, ^29^, ^30^, ^31^, ^32^, ^33^ Finally, according to the simulations with the final model, the number of patients with rituximab concentration below 4 mg/L at 3 months after the first maintenance dose of the 500 mg Q6M regimen was 16%, which is coherent with the values reported in MAINRITSAN2 phase 3 trial (18%).^21^

An indirect response model satisfactorily described gammaglobulin data over time. The mean estimate of baseline gammaglobulin levels was 9.35 g/L which is consistent with values reported in a real-world study including 98 patients with AAV (10.4 g/L).^20^ The mean estimation of first-order degradation rate constant of gammaglobulins (k_out,GG_) was 0.0034 day^−1^ which is lower than the elimination half-life of immunoglobulins in humans (18–26 days, i.e. k_out,GG_ of 0.039–0.027 day^−1^).^19^ However, as PK/PD analysis aims to represent complex biological processes by simplified mathematical models, k_out,GG_ in our model might represent not only the elimination of gammaglobulins but also other physiological phenomena impacting their circulating levels. According to simulations of the 375 mg/m^2^ Q1W induction regimen, the median gammaglobulin value decreased from 9.3 g/L at baseline to 7.0 g/L at start of maintenance phase (i.e. at 6 months), representing a median decrease of 25%. This is consistent with the study of Liberatore et al. where a decrease from 10.3 g/L to 7.6 g/L was observed (median decrease of 25%) in 98 patients with AAV treated with rituximab.^20^ In our simulations with 375 mg/m^2^ Q1W induction regimen, 34% of patients had gammaglobulin levels <6 g/L at month 6, which is consistent with the number reported in Liberatore et al. (31.4%).^20^ Finally, simulated profiles of gammaglobulins during maintenance phase at 500 mg Q6M regimen were consistent with values observed before each maintenance dose in the study by Liberatore et al.^20^ further validating our model.

MPO-ANCA and PR3-ANCA concentration–time data were described jointly using a previously proposed model for ANCA.^19^ The mean estimates of model parameters in our study are consistent with those from the RAVE trial^19^ although in that study, separate parameters were estimated for MPO-ANCA and PR3-ANCA. Mean estimated MTT of 31.9 days for ANCA is consistent with physiological half-life of immunoglobulins.^19^ The mean estimate of baseline ANCA level (BASE_ANCA_) was 75.6 U/mL and was slightly lower than values estimated in the PK/PD analysis of the RAVE trial (95.6 U/mL and 138 U/mL for MPO-ANCA and PR3-ANCA, respectively) which might be due to different characteristics of the studied populations. Furthermore, the number of patients achieving negative ANCA at 6 months after induction with 375 mg/kg Q1W in our simulations (46% and 50% for PR3-and MPO-ANCA, respectively), is coherent with the values observed in RAVE clinical trial (47% and 40% of patients with PR3-and MPO-ANCA, respectively),^19^ which validates our final PK/PD model.

The achievement of serological remission within 6 months of the first induction dose has recently been correlated with a reduced risk of subsequent relapse, suggesting that it may be a surrogate marker of treatment efficacy.^13^ In the present study, simulations showed that both induction regimens (375 mg/m^2^ Q1W and 1000 mg D0-D14) resulted in a similar number of patients achieving serological remission at 6 months. These results are consistent with the comparable clinical efficacy of both induction regimens reported in a meta-analysis.^7^ Complete remission was achieved in 85% (95% confidence interval [CI]: 70–96) and 91% (95% CI: 79–99) of patients treated with the 375 mg/m^2^ Q1W and 1000 mg D0-D14 regimens, respectively. In terms of safety, both regimens were associated with a similar percent of serious infections (12% in both regimens), which is consistent with our simulations for hypogammaglobulinaemia (<6 g/L) incidence at month 6 between the two regimens (34% for both). Although a prospective study comparing these two induction regimens has not been performed, our PK/PD analysis support the choice of either regimen. However, in terms of patient quality of life and treatment costs, 1000 mg D0-D14 may be preferred as it is associated with fewer hospital visits and therefore lower treatment costs (€6624 per patient for 1000 mg D0-D14 versus €11,264 for 375 mg/m^2^ Q1W). In this context, we selected the 1000 mg D10-D14 induction regimen for further simulations of maintenance regimens.

For the maintenance regimens, simulations showed that more patients achieved negative MPO-ANCA and PR3-ANCA at month 24 with RITAZAREM regimen (1000 mg Q4M) than with MAINRITSAN regimen (500 mg Q6M). This was primarily due to more frequent infusions in the 1000 mg Q4M regimen rather than higher dose. Indeed, similar number of patients achieved MPO-ANCA negativity at month 24 with 1000 mg Q4M than 500 mg Q4M (79% versus 75%, respectively). The difference was slightly more important for PR3-ANCA (77% versus 65%, respectively). The comparison between the 500 mg Q4M and 500 mg Q6M regimens showed the superiority of the 500 mg Q4M regimen in terms of serological remission for both PR3-ANCA (65% versus 46%) and MPO-ANCA (75% versus 59%). As increasing the dose and/or frequency of administration was associated with a higher number of patients with hypogammaglobulinaemia, the results of the simulations were compared in terms of the risk-benefit ratio by calculating the utility score.^22^ When considering serum gammaglobulins <6 g/L as safety endpoint, 1000 mg Q4M regimen had significantly lower utility score than 500 mg Q6M (start at month 4 or 6), and a tendency was observed for 500 mg Q4M. Importantly, 500 mg Q4M regimen was associated with comparable utility to RITAZAREM regimen (1000 mg Q4W), but with lower treatment costs. When greater weight is assigned to efficacy, RITAZAREM regimen has a significantly higher utility score than MAINRITSAN regimen in patients with PR3-ANCA. Further selection of the maintenance regimen should be based on individual patient response. In cases of suboptimal efficacy, more frequent dosing (i.e. Q4M) may be preferred over Q6M, or a higher dose (1000 mg) may be considered, if the patient has a favourable toxicity profile. This is further supported by a higher percentage of patients with rituximab concentration >4 mg/L at 3 months after the first maintenance dose^21^ with RITAZAREM regimen (94%) compared to regimens with a 500 mg dose (84–87%, Table 4). Indeed, a rituximab concentration <4 mg/L at 3 months after the first maintenance dose was identified as an independent risk factor of major relapse at month 28 in the cohort of MAINRITSAN2 phase 3 trial.^21^ Monitoring of rituximab plasma concentration 3 months after the first maintenance dose could be useful in individualising the dosing regimen in underexposed patients.

This study has several limitations. Other factors such as the presence of anti-drug antibodies (ADA) may also affect the PK^33^ and efficacy/safety of rituximab,^35^ but were not available in our study. Recently, Hartinger et al. reported a 15% decrease in rituximab CL in ADA-positive patients with autoimmune glomerular diseases.^33^ Future studies should collect neutralising and total ADA of rituximab for a more thorough interpretation of their impact on PK/PD relationship.

This study relies primarily on serological biomarkers, without correlation to clinical data (relapses, severe infections). However, previous studies have reported associations between ANCA and gammaglobulins with clinical outcomes. McDermott et al. showed in 506 patients with AAV that those achieving negative ANCA titre within 180 days after induction with rituximab had lower risk of relapse.^13^ Fussner et al. reported that PR3-ANCA reappearance or increase after initial remission was associated with relapses in patients with AAV treated with rituximab.^36^ Finally, PR3-ANCA positivity and azathioprine arm were independently associated with a higher risk of relapse in a trial comparing rituximab with azathioprine to maintain remission in 115 patients with AAV.^37^ Regarding the correlation between gammaglobulin levels and the risk of severe infections, a study involving 98 patients treated with rituximab for AAV found that severe infection-free survival was significantly poorer in patients with both gammaglobulin levels <6 g/L and gammaglobulin decline >25%.^20^

CD19+ counts were not included in this PK/PD analysis as a potential marker of efficacy in addition to ANCA. CD19+ counts have never been studied as a surrogate marker of efficacy, but only as a marker that may help to determine the most appropriate time to administer a new dose of rituximab.^9^ Nevertheless, the results of the MAINRITSAN3 trial showed that none of the patients with both ANCA and CD19+ B-cell negativity experienced a relapse.^38^ Although longitudinal data on CD19+ counts were collected as part of this study, it could not be satisfactorily described by any of the tested PK/PD models (depleted CD19+ counts throughout treatment). Further PK/PD studies that include both ANCA and CD19+ as markers of rituximab efficacy in AAV are needed.

Finally, the results from this study need to be further confirmed in a prospective clinical trial, where the selection of maintenance regimens to be tested can be based on simulations from our PK/PD model. In addition, this PK/PD analysis is restricted to treatment period and makes no assumptions regarding future patient's health transitions, whereas the pharmacoeconomic analysis includes direct costs (drug and administration) but not long-term or indirect costs. The results can inform broader economic analyses such as health state transition modelling.

In conclusion, the PK/PD model-based simulations show that the two rituximab induction regimens are similar in terms of efficacy and safety. In the maintenance phase, 500 mg Q6M (start at month 4 or 6), had a significantly higher utility score than 1000 mg Q4M, due to lower number of patients with hypogammaglobulinaemia. However, when greater weight is assigned to efficacy over safety, the 1000 mg Q4M regimen may be preferred over the 500 mg Q6M in patients with PR3-ANCA. This study may help inform patients and clinicians when weighing the risks and benefits of rituximab treatment.

## Contributors

**Blaise Pasquiers**: Accessed and verified the underlying data; Data curation; Formal analysis; Writing–original draft; Final approval of the version to be submitted.

**Benoit Blanchet:** Accessed and verified the underlying data; Supervision; Methodology; Writing–review & editing; Conceptualisation; Validation; Final approval of the version to be submitted.

**Xavier Puéchal:** Conceptualisation; Methodology; Supervision; Writing–review & editing; Investigation; Validation; Final approval of the version to be submitted.

**Xavier Declèves:** Writing–review & editing; Methodology; Supervision; Final approval of the version to be submitted.

**Pascal Cohen:** Investigation; Writing–review & editing; Final approval of the version to be submitted.

**Claire Goulvestre:** Writing–review & editing; Investigation; Final approval of the version to be submitted.

**Marion Casadevall:** Writing–review & editing; Investigation; Final approval of the version to be submitted.

**Inès Benhabiles:** Investigation; Final approval of the version to be submitted.

**Michel Vidal:** Writing–review & editing; Investigation; Final approval of the version to be submitted.

**David Ternant:** Methodology; Supervision; Writing–review & editing; Validation; Final approval of the version to be submitted.

**Benjamin Terrier:** Writing–review & editing; Writing–original draft; Conceptualisation; Investigation; Supervision; Methodology; Validation; Final approval of the version to be submitted.

**Alicja Puszkiel:** Accessed and verified the underlying data; Methodology; Supervision; Writing–original draft; Formal analysis; Final approval of the version to be submitted.

## Data sharing statement

The datasets generated and analysed during the current study are available from the corresponding author upon reasonable request. Requests for data sharing should be directed to alicja.puszkiel@aphp.fr.

## Declaration of interests

B.B. received consultancy and speaking fees from Bristol Myers Squibb, GlaxoSmithKline, Eisai, Ipsen, Astra Zeneca, Pierre Fabre and Pfizer.

X.P. has received consultancy and speaking fees from Boehringer Ingelheim and GlaxoSmithKline, and has been an investigator in academic trials in ANCA-associated vasculitis where rituximab was provided by Roche.

D.T. received speaking fees from Novartis, Sanofi, Lundbeck, and Amgen.

B.T. has received consultancy and speaking fees from AstraZeneca, GlaxoSmithKline, Boehringer Ingelheim, Novartis, CSF Vifor.

A.P. received speaking fees from Bristol Myers Squibb, Pierre Fabre Oncology and Eisai.

The remaining authors declare no conflict of interest.
