# Supplementary material for: Comparison of rituximab induction and maintenance regimens in anti-neutrophil cytoplasmic antibodies (ANCA)-associated vasculitis: PK/PD modelling of ANCA and gammaglobulin levels in real-world patients
Source: eBioMedicine. 2025 Nov 3;121:105989. doi: 10.1016/j.ebiom.2025.105989 (PMC12677076; doi:10.1016/j.ebiom.2025.105989)
Supplement: Supplementary Material [file mmc1.docx]

Electronic Supplementary Material to:

**Comparison of rituximab induction and maintenance regimens in anti-neutrophil cytoplasmic antibodies (ANCA)-associated vasculitis: PK/PD modelling of ANCA and gammaglobulin levels in real-world patients**

Blaise Pasquiers, PharmD, PhD^1,2^, Benoit Blanchet, PharmD, PhD^3,4^, Xavier Puéchal, MD, PhD^5,6^, Xavier Declèves, PharmD, PhD^1,3^, Pascal Cohen, MD^5^, Claire Goulvestre, PharmD, PhD^7^, Marion Casadevall, MD^5^, Inès Benhabiles, PharmD^3^, Michel Vidal, PharmD, PhD^3,4^, David Ternant, PharmD, PhD^8,9^, Benjamin Terrier, MD, PhD^5,6^, Alicja Puszkiel, PharmD, PhD^1,3^

1. Université Paris Cité, INSERM, Optimisation thérapeutique en neuropharmacologie OTEN U1144, 75006, Paris, France
2. PhinC Development, 36 rue Victor Basch, Massy, France
3. Biologie du Médicament – Toxicologie, Cochin University Hospital, AP-HP, Paris, France
4. Université Paris Cité, Faculty of Pharmacy, CNRS UMR8038, Inserm U1268, CARPEM, Paris, France
5. Department of Internal Medicine, National Referral Center for Rare Systemic Autoimmune Diseases, Hôpital Cochin, Assistance Publique-Hôpitaux de Paris, Paris, France
6. Université Paris Cité, F-75006 Paris, France
7. Laboratory of Immunology, Cochin University Hospital, AP-HP, Paris, France
8. Université de Tours, EA 4245 T2I, Tours, France
9. Service de Pharmacologie Médicale, CHRU de Tours, Tours, France

**Supplementary Material S1.** Handling data below lower (BLOQ) or above upper (ULOQ) limit of quantification

Rituximab BLOQ data and ANCA BLOQ or ULOQ data were included in the analysis using left-censoring in Monolix® Software (equivalent to M3 in NONMEM) which allows to include these samples in the computation of the likelihood. ^1^ Indeed, the likelihood of the BLOQ sample assumes that the value is less than the lower limit of quantification (LLOQ) whereas for samples above upper limit of quantification (ULOQ), it assumes that the value is higher than ULOQ. In all the goodness-of-fit plots, the censored data refers to data included in the analysis with left-censoring.

**Supplementary Material S2.** Details on the development of structural and statistical models.

The PK/PD modelling analysis was performed using population approach (nonlinear mixed effects modelling). In this approach, data from all patients are analysed simultaneously. First, structural models describing the biological phenomena driving the observed concentration-time profiles for rituximab, gamma globulins and ANCA were developed based on physiology, plausibility and precision of parameter estimates and statistical criteria. The mean estimated parameter values represent a typical patient profile. An interindividual variability term (interindividual variance) is added on structural parameters to account for the variability of the biological phenomenon in the studied population and evaluate the impact of potential biological, demographic or disease-related factors (covariates) on model parameters and explain the observed variability of the parameters in the studied population. The population approach allows therefore to estimate parameters at the population level (mean values of estimated parameters and their distribution in the studied population); inclusion of interindividual variability term allows to derive individual parameter values.

# Structural model

## Rituximab PK model

The elimination of rituximab is mediated by non-saturable catabolism and by the formation of rituximab-CD20 complexes. These complexes are recognised and cleared by the immune system and this mechanism is responsible for target-mediated drug disposition (TMDD) of rituximab, a saturable non-linear mechanism. This non-linear elimination clearance (CL) of rituximab is dependent on the underlaying disease ^2^ and has been described in non-Hodgkin’s lymphoma ^3^ and large B-cell lymphoma. ^4^ In our study, one- and two-compartment models with linear elimination from the central compartment with or without a non-linear or a time-varying elimination clearance (CL) were tested. Time-dependency on CL was tested using a hyperbolic or sigmoidal function as previously proposed by Petitcollin *et al*. ^5^:

$${CL}_{TD}={CL}_{init}\cdot exp( \frac{E_{max}\cdot{TIME}^{\gamma}}{T_{50}^{\gamma}+{TIME}^{\gamma}})$$

Where CL_TD_ is the time-dependent clearance, CL_init_ is the initial clearance (at treatment start), E_max_ is the maximal change in CL_init_ in time, T_50_ is the time at which the half of maximal change in CL is achieved and γ is the Hill coefficient describing the sigmoidicity of the relationship between E_max_ and time. In the hyperbolic model γ was fixed to 1.

## Gammaglobulins PK/PD model

The structural model for gammaglobulins consisted of a turnover model with zero-order production and first-order elimination of gammaglobulins. Since rituximab decreases the number of B cells and therefore, the synthesis of gammaglobulins, the model assumes that plasma concentration of rituximab inhibited the zero-order synthesis of gammaglobulins (k_in,GG_) according to the following equation:

$$\frac{dGG}{dt} = k_{in,GG}\cdot\left( 1-E_{drug} \right)- k_{out,GG}\cdot GG$$

$$GG\left( 0 \right)={BASE}_{GG}$$

Where *GG* is the gammaglobulin concentration, *k_in_* is the zero-order gammaglobulin synthesis constant, *k_out_* is the first-order gammaglobulin elimination rate constant, *E_drug_* is the effect of the drug and BASE_GG_ is the baseline serum gammaglobulins concentration. The model was parameterised in terms of k_out,GG_ and BASE_GG_ (k_in,GG_/k_out,GG_). Both linear and I_max_ functions were tested to describe the drug effect as follows:

$E_{drug} = E\cdot C_{plasma}$ (Linear)$E_{drug} = \frac{I_{max}\cdot C_{plasma}}{{IC}_{50}+C_{plasma}}$ (I_max_)

Where *E* is the slope of the drug effect in the linear model, *I_max_* is the maximum inhibition of gammaglobulin synthesis by rituximab (fixed to 1), *IC_50_* is the rituximab concentration that produces 50% of the maximum inhibition, *C_plasma_* is the plasma rituximab concentration.

## ANCA PK/PD model

Several structural models were tested including an indirect response model with a turnover of ANCA concentrations, a Friberg model. ^6^ This model was firstly developed to describe hematologic cell evolution over time but since then, has been adapted to describe maturation and proliferation of other blood components including platelets ^7^ and ANCA. ^2^ The structural model developed by Bensalem *et al*. based on data from the RAVE trial ^2^ is depicted in **Figure 1** in the manuscript. The model consisted of two compartments: first compartment represents ANCA production compartment and the second represents observed ANCA in the blood (circulating) compartment. A negative feedback mechanism from the observed ANCA compartment to the production compartment is used to describe the rebound of cells compared with the baseline value. Rituximab inhibits ANCA production by targeting CD20 on B cells, which was translated in the PK/PD model by estimating the inhibitory effect of rituximab on ANCA production in the deep (production) compartment. The model is defined using the following equations:

$$\frac{d{ANCA}_{prod}}{dt} = k_{prod}\cdot{{ANCA}_{prod}\cdot(1-E_{drug})\cdot\left( \frac{{ANCA}_{blood\_0}}{{ANCA}_{blood}} \right)}^{{Gamma}_{ANCA}}-k_{tr}\cdot{ANCA}_{prod}$$

$$\frac{d{ANCA}_{blood}}{dt} = k_{tr}\cdot{ANCA}_{prod}-k_{rem}\cdot{ANCA}_{blood}$$

$${ANCA}_{prod}\left( 0 \right)={ANCA}_{blood}\left( 0 \right)={BASE}_{ANCA}$$

$$k_{tr}= k_{prod}=k_{rem}$$

Where *ANCA_prod_* is the amount of ANCA in the deep (production) compartment, *ANCA_blood_* is the amount of ANCA in the blood (circulation) compartment, *k_prod_* is the first-order ANCA production rate constant, *k_tr_* is the first-order transit rate constant and is the inverse of the mean transit time (MTT=1/k_tr_). *k_rem_* is the first-order rate constant describing ANCA removal from the blood compartment. *Gamma_ANCA_* is the power of the negative feedback from the blood compartment to the deep compartment.

Both linear and I_max_ functions (with I_max_ fixed to 1) were tested to describe the inhibitory effect of rituximab on ANCA production (*E_drug_*).

# Statistical model

Statistical model allows to describe interindividual variability in model parameters and residual error, i.e. the difference between the observed and model-predicted concentrations.

## Interindividual variability

PK and PD parameters were assumed to follow a log-normal distribution according to the following equation:

$$\theta_{i}=\theta_{pop}\cdot e^{\eta_{i}}$$

Where θ_i_ is the value of the parameter for *i*th subject, θ_pop_ is the population estimated value for this parameter and *η_i_* is the individual value of the random effect associated with that parameter describing the difference between the mean (θ_pop_) value of the model parameter and the individual value of that parameter for *i*th subject (θ_i_).

The inclusion of interindividual variability term was tested on all the parameters. If the interindividual variability could not be reliably estimated (implausible value of parameter, high imprecision of estimation, i.e. high relative standard error (RSE%), no improvement in corrected Bayesian Information Criteria (BICc)), it was fixed to 0 (i.e. it was considered that all the individuals had the same value of that parameter).

## Residual variability

Proportional and combined error models were tested to describe the residual unexplained variability and its selection was based on BICc. A separate residual error was estimated for PK, gammaglobulin and ANCA data. In the ANCA PK/PD model, both separate and shared residual error was tested for MPO-ANCA and PR3-ANCA.

**Supplementary Material S3.** Details of the covariate analysis.

The effect of covariates was evaluated using the following equations:

$\theta_{i}=\theta_{pop}\cdot\left( \frac{{cov}_{i}}{{cov}_{weighted mean}} \right)^{\theta_{cov}}\cdot e^{\eta_{i}}$ (Continuous covariate)

$\theta_{i}=\theta_{pop} \cdot e^{\theta_{cov}\times{cov}_{i}}\cdot e^{\eta_{i}}$ (Categorical covariate)

$${cov}_{weighted mean}=exp(\sum_{i} \frac{{nbObs}_{i}}{nbObs}\log\left( {cov}_{i} \right))$$

Where *θ_i_* is the individual value for the parameter, *θ_pop_* is the population value for this parameter, *cov_i_* is the individual covariate value, *cov_weighted mean_* is the weighted mean of the covariate in the studied population, *θ_cov_* is the effect of the covariate on the parameter, *η_i_* is the individual value of the random effect associated to the parameter describing the difference between *θ_pop_* and *θ_I_,* *nbObs_i_* is the number of observations for the *i*th subject and *nbObs* is the total number of observations. In case of missing continuous covariates, data was imputed with the population mean. Missing categorical data were imputed with the most frequent category.

Covariate analysis was performed according to COSSAC method in Monolix. ^8^ For each parameter-covariate relationship, Pearson’s correlation test (continuous covariates) or Anova test (categorical covariates) was performed in the base model. Starting with the most significant correlation p-value, all significant covariates were included in the model one by one leading to the full model. Then, the backward selection was performed in which covariates with the least significant correlation p-value were removed from the model one by one until all non-significant covariates were excluded. Selection of covariates was based on improvement in BICc, Wald test with p-value < 0.01 in the backward step, stability and plausibility of parameter estimates and decrease in IIV of the associated parameter.

**Supplementary Material S4.** Details regarding simulations of induction and maintenance regimens.

The mean estimates of population parameters, covariate effects and random effects (interindividual variability and residual error) from the final models were used in simulations. Rituximab, serum gammaglobulin, MPO-ANCA and PR3-ANCA concentrations were simulated for different induction and maintenance regimens (n = 1000 patients for each scenario).


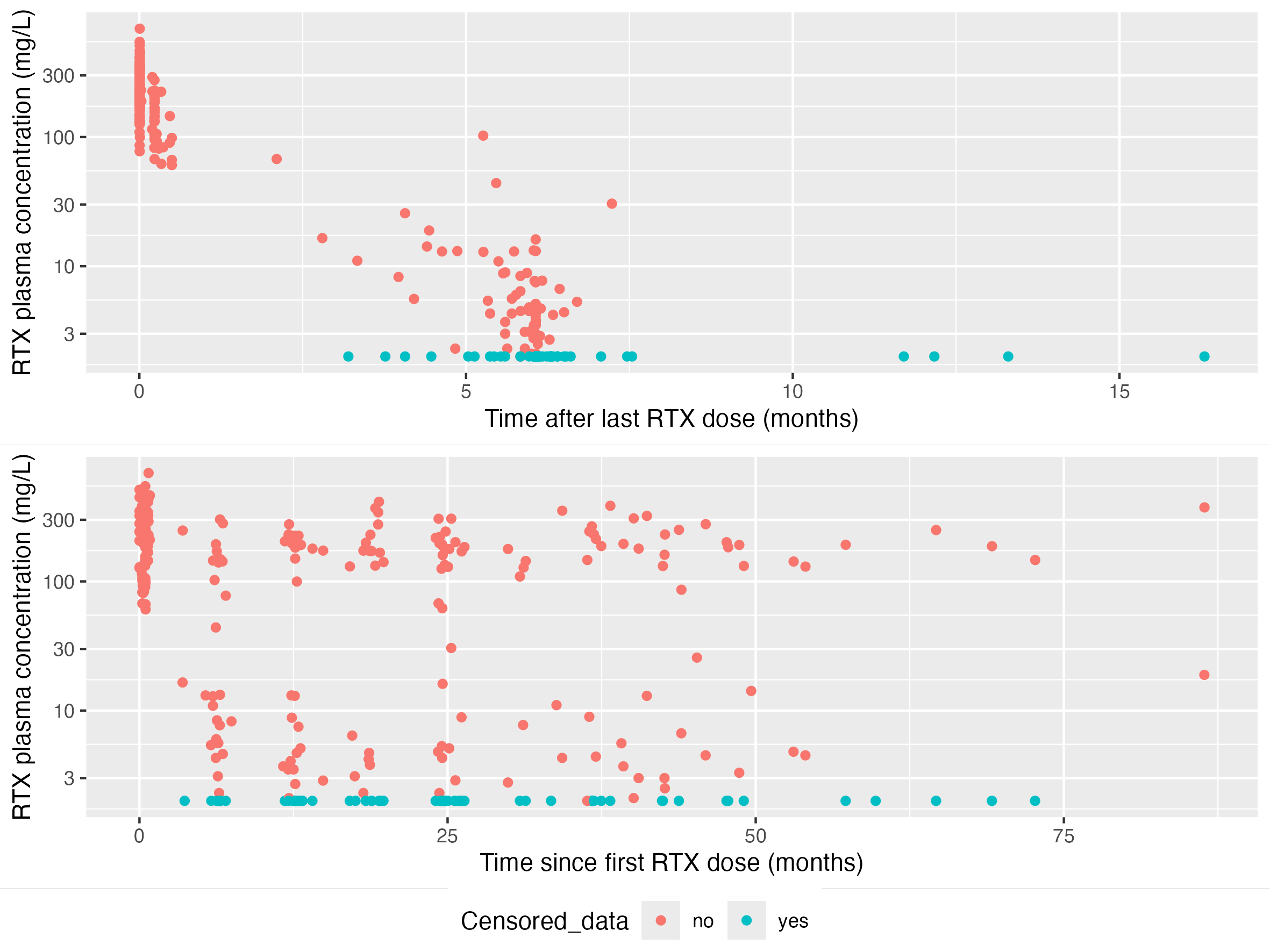


**Figure S1.** Plasma rituximab (RTX) concentrations versus time after last rituximab dose and time since first rituximab infusion. Censored data represent data below lower limit of quantification (BLOQ), included in the analysis using left-censoring method.


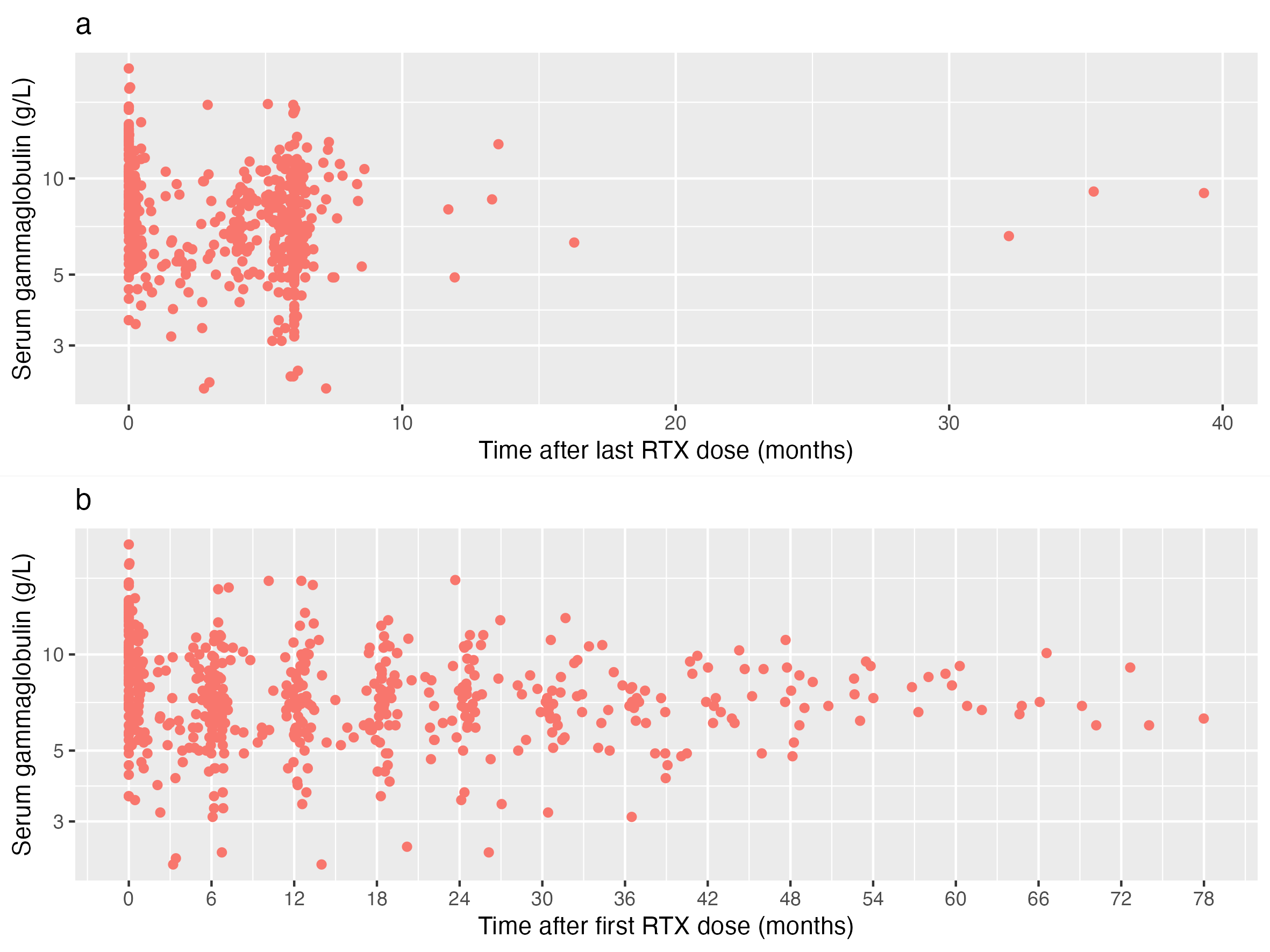


**Figure S2.** Serum gammaglobulin levels versus (a) time after last rituximab dose and (b) time since first rituximab infusion.


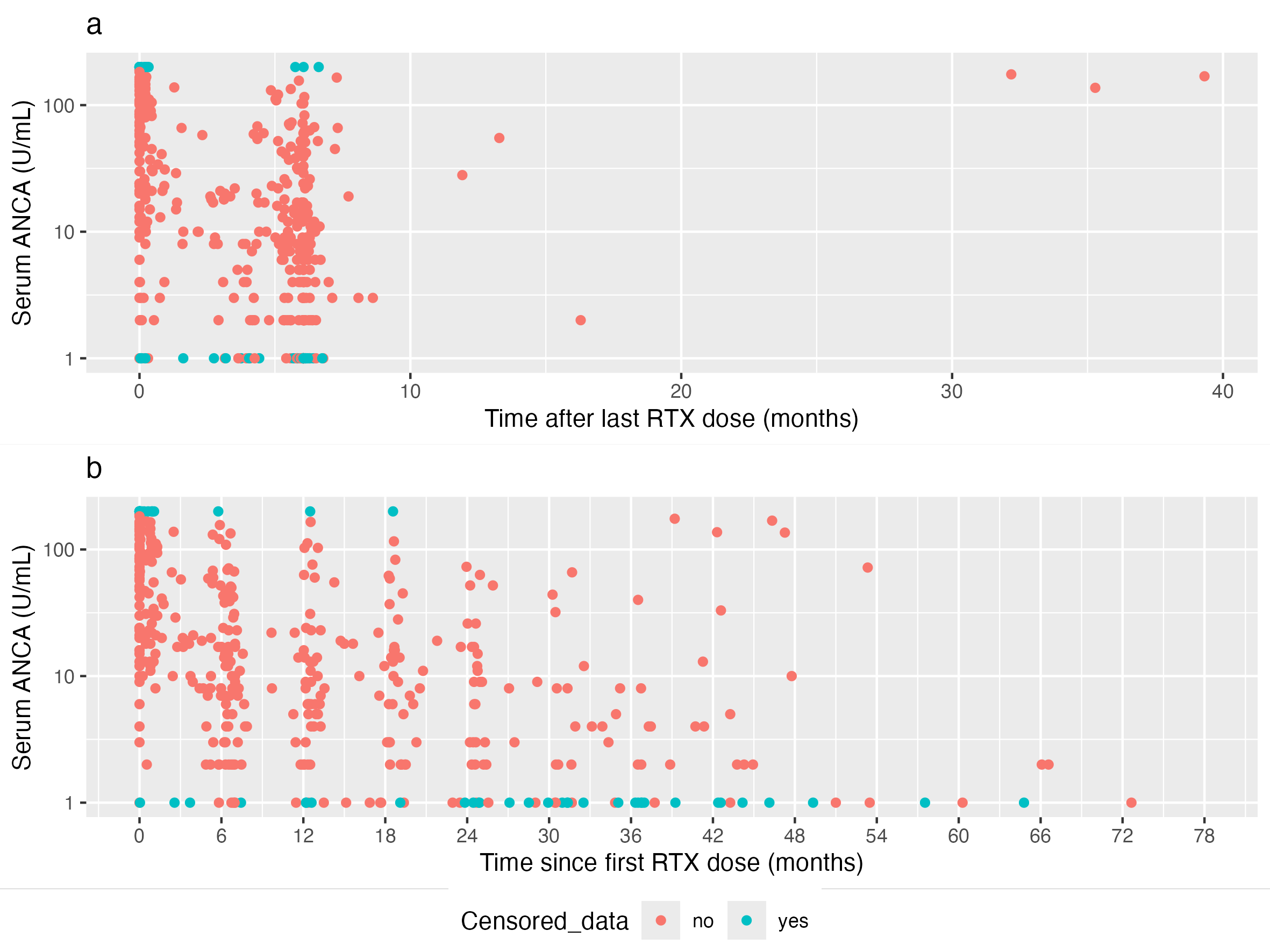


**Figure S3.** Serum ANCA levels versus (a) time after last rituximab dose and (b) time since first rituximab infusion. Censored data represent data below lower limit of quantification (BLOQ) or above upper limit of quantification (ULOQ), included in the analysis using left-censoring method.


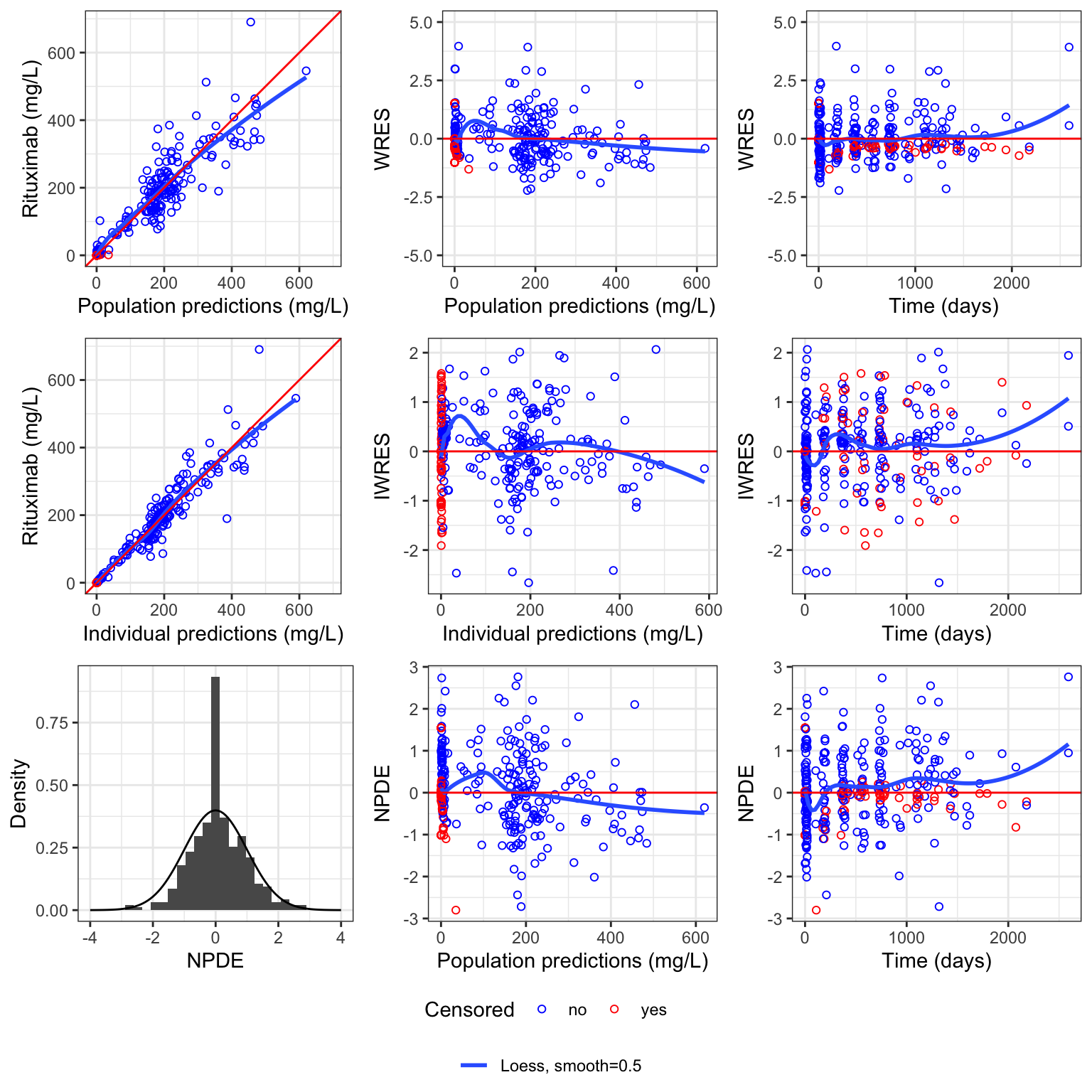
**Figure S4.** Goodness of fit plots of the final PK model of rituximab. WRES, weighted residuals; IWRES, individual weighted residuals; NPDE, normalized prediction distribution error. Censored data represents concentrations below lower limit of quantification which were included in the analysis using left-censoring method.


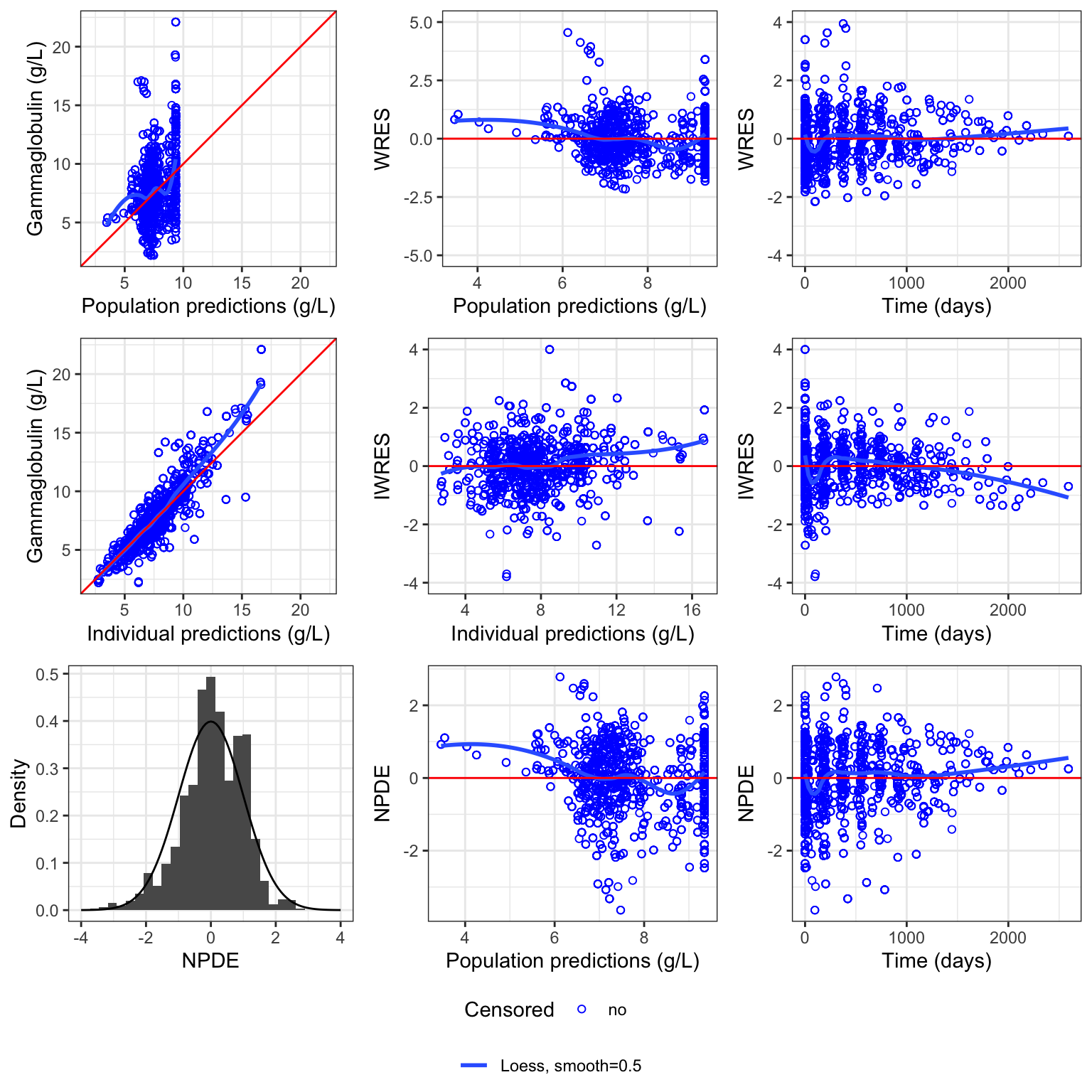
**Figure S5.** Goodness of fit plots of the final PK model of serum gammaglobulin levels. WRES, weighted residuals; IWRES, individual weighted residuals; NPDE, normalized prediction distribution error.


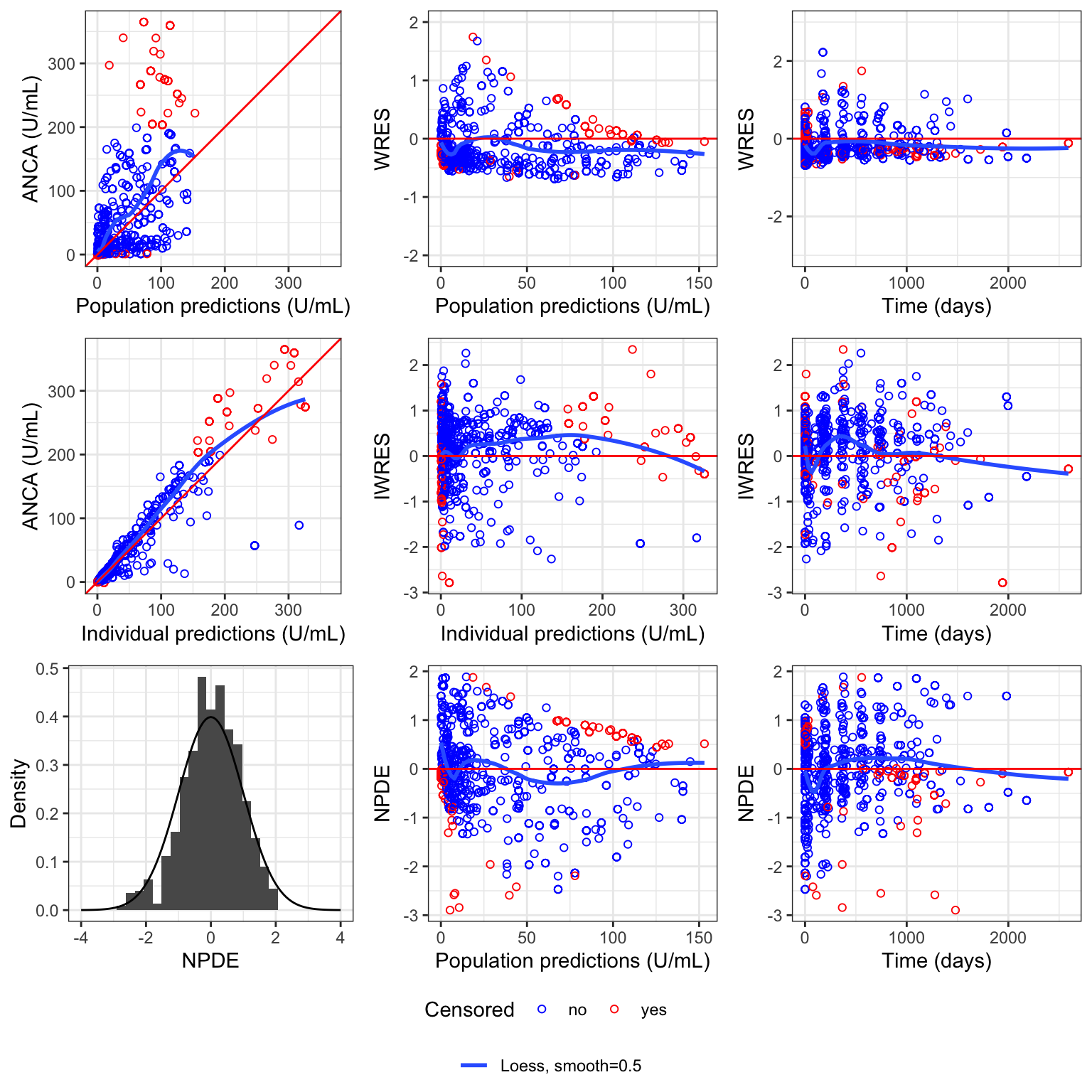


**Figure S6.** Goodness of fit plots of the final PK model of MPO-ANCA and PR3-ANCA (data were modelled together). WRES, weighted residuals; IWRES, individual weighted residuals; NPDE, normalized prediction distribution error. Censored data represents concentrations below lower limit of quantification or above upper limit of quantification which were included in the analysis using left-censoring method.


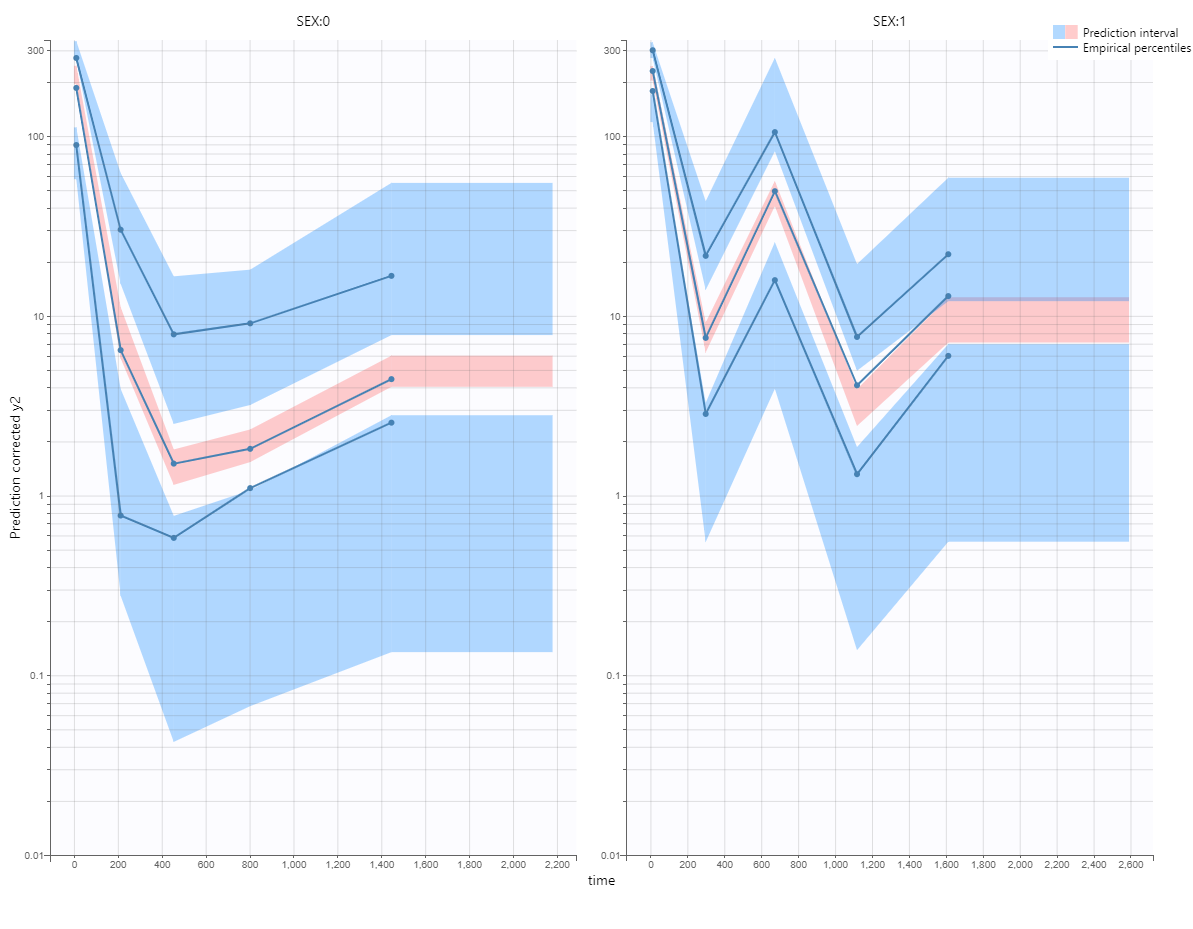


**Figure S7.** pcVPC of the final rituximab model (plasma rituximab concentrations on log scale versus time since first infusion) stratified on SEX (0 = male, 1 = female). Solid lines represent the empirical percentiles (5^th^, 50^th^ and 95^th^) and areas represent the 90% prediction interval around 5^th^, 50^th^ and 95^th^ percentiles of the simulated data.

**
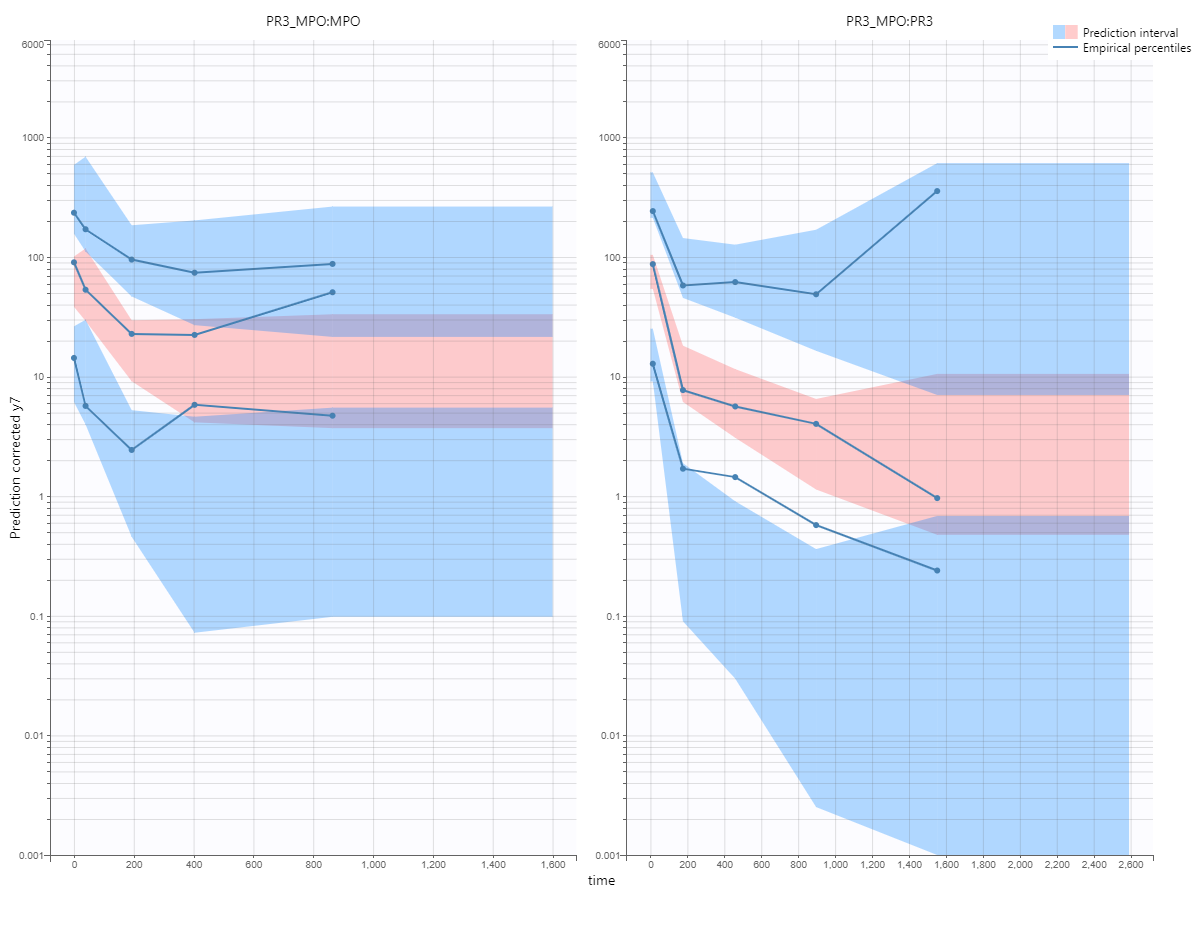
**

**Figure S8.** pcVPC of the final ANCA PK/PD model (ANCA concentrations on log scale versus time since first infusion) stratified on type of ANCA (MPO and PR3). Solid lines represent the empirical percentiles (5^th^, 50^th^ and 95^th^) and areas represent the 90% prediction interval around 5^th^, 50^th^ and 95^th^ percentiles of the simulated data.

**Supplementary Material S5.** Equations of the final covariate model.

${CL}_{i}={CL}_{pop}\cdot\left( \frac{{age}_{i}}{52} \right)^{-0.33}\cdot e^{-0.23 if SEX=1} \cdot e^{\eta_{i}}$

Where *CL_i_* is the individual value of rituximab clearance, *CL_pop_* is the population value of rituximab clearance, *age_i_* is the individual age value, *η_i_* is the individual value of the random effect associated to the parameter describing the difference between *CL_pop_* and *CL_i._*

${V1}_{i}={V1}_{pop}\cdot\left( \frac{{BW}_{i}}{68} \right)^{0.71}\cdot e^{\eta_{i}}$

Where *V1_i_* is the individual value of rituximab central volume of distribution, *V1_pop_* is the population value of that parameter, BW*_i_* is the individual body weight value, *η_i_* is the individual value of the random effect associated to the parameter describing the difference between V1*_pop_* and *V1_i._*

${BASE}_{ANCAi}={BASE}_{ANCApop}\cdot\left( \frac{{CRP}_{i}}{11} \right)^{0.21}\cdot e^{\eta_{i}}$

Where *BASE_ANCAi_* is the individual value of ANCA level at baseline, *BASE_ANCApop_* is the population value of that parameter, *η_i_* is the individual value of the random effect associated to the parameter describing the difference between *BASE_ANCApop_* and *BASE_ANCAi._*

${EC50}_{i}={EC50}_{pop}\cdot e^{-0.96 if ANCA=PR3}\cdot e^{\eta_{i}}$

Where *EC50_i_* is the individual value of rituximab half-maximal inhibitory concentration on ANCA synthesis, *EC50_pop_* is the population value of that parameter, *η_i_* is the individual value of the random effect associated to the parameter describing the difference between *EC50_pop_* and *EC50_i._*

**Table S1.** Percent decrease from baseline in ANCA levels for induction and maintenance regimens. Data are expressed as percentage of simulated patients (n = 1000).

|  | MPO-ANCA | PR3-ANCA |
| --- | --- | --- |
|  | **Median decrease from baseline [5^th^-95^th^ percentile]** | |
| Induction regimens |  | |
|  | **At 6 months after first induction dose** | |
| 375 mg/m^2^ every week for 4 weeks | 69% [20 – 98] | 66% [17 – 98] |
| 1000 mg Day 0 – Day 14 | 64% [17 – 98] | 58% [16 – 96] |
| Maintenance regimens |  | |
|  | **At 12 months after first induction dose** | |
| 500 mg Q6M (MAINRITSAN regimen) | 72% [23 – 99] | 67% [20 – 99] |
| 500 mg Q6M, start at month 4 | 79% [26 – 100] | 72% [26 – 99] |
| 500 mg Q4M | 82% [27 – 100] | 74% [24 – 100] |
| 1000 mg Q4M (RITAZAREM regimen) | 81% [33 – 100] | 83% [30 – 100] |
|  | **At 24 months after first induction dose** | |
| 500 mg Q6M (MAINRITSAN regimen) | 80% [31 – 100] | 75% [25 – 99] |
| 500 mg Q6M, start at month 4 | 84% [32 – 100] | 78% [28 – 100] |
| 500 mg Q4M | 90% [38 – 100] | 83% [32 – 100] |
| 1000 mg Q4M (RITAZAREM regimen) | 94% [50 – 100] | 94% [45 – 100] |

MPO-ANCA, myeloperoxidase antineutrophil cytoplasmic antibodies; PR3-ANCA, proteinase 3 antineutrophil cytoplasmic antibodies; Q4M, every 4 months; Q6M, every 6 months.

**Table S2.** Cost of different induction and maintenance phase regimens considering rituximab price (€1.412 per mg) and the price of daily care unit in France (€1,900).

| Treatment phase | Regimen | Total number of daily care unit visits | Rituximab total dose (mg) | Total price per patient (€) |
| --- | --- | --- | --- | --- |
| Induction | 375 mg/m^2^ every week for 4 weeks^a^ | 4 | 2,595 | 11,264 |
|  | 1000 mg D0-D14 | 2 | 2,000 | 6,624 |
| Maintenance | 500 mg Q6M (MAINRITSAN) | 4 | 2,000 | 10,424 |
|  | 500 mg Q6M start at 4^th^ month | 4 | 2,000 | 10,424 |
|  | 500 mg Q4M | 5 | 2,500 | 13,030 |
|  | 1000 mg Q4M (RITAZAREM) | 5 | 5,000 | 16,560 |

D0-D14, day 0 and day 14, Q4M, every 4 months; Q6M, every 6 months;

^a^ cost calculated for a patient with body surface area of 1.73 m^2^


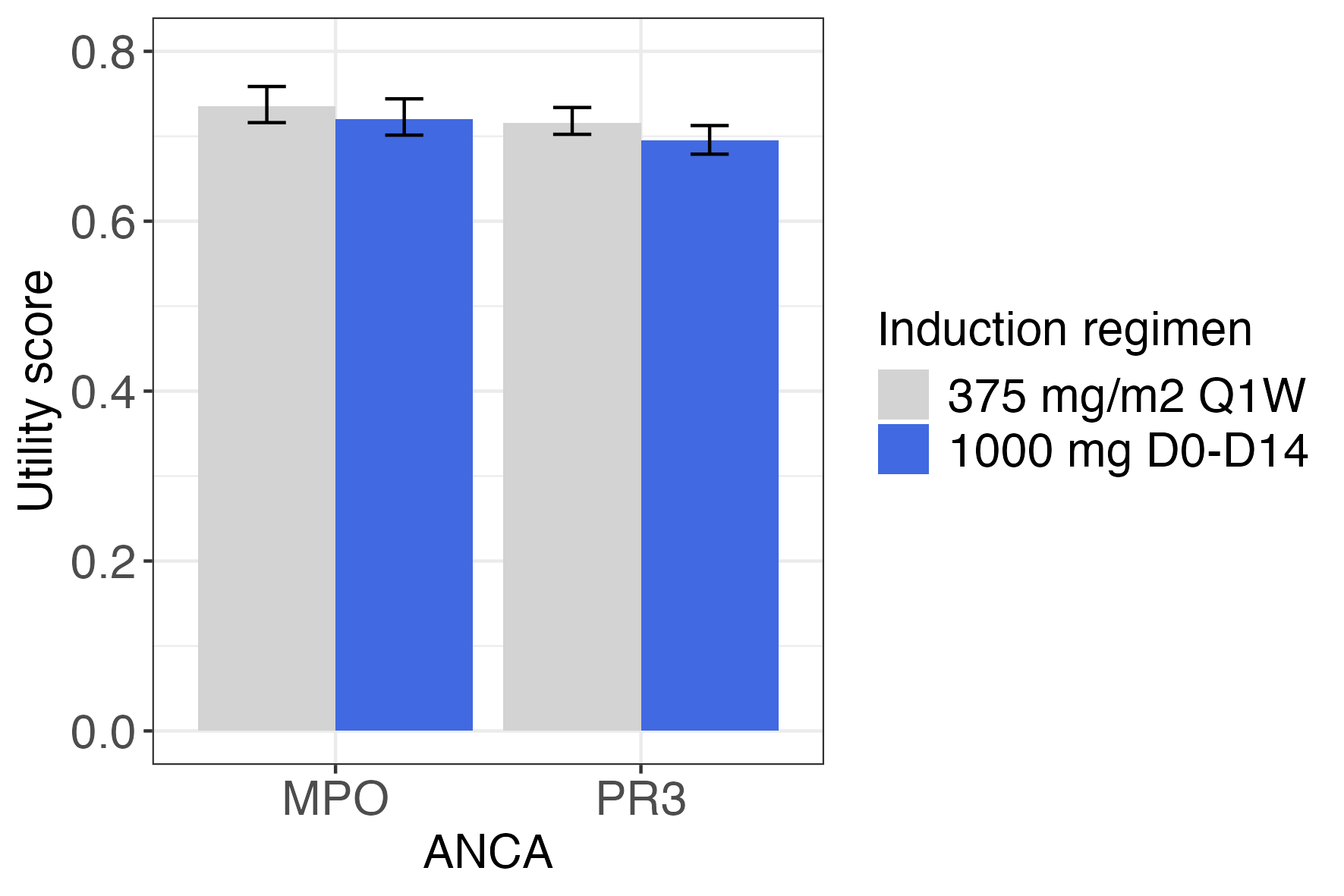


**Figure S9.** Clinical utility score for rituximab induction regimens with weighting allocation of 0.5/0.5 (efficacy/safety) considering patients with as serum gammaglobulins < 4 g/L. Data are represented as median of 1000 simulations, the bars represent 90% prediction intervals.


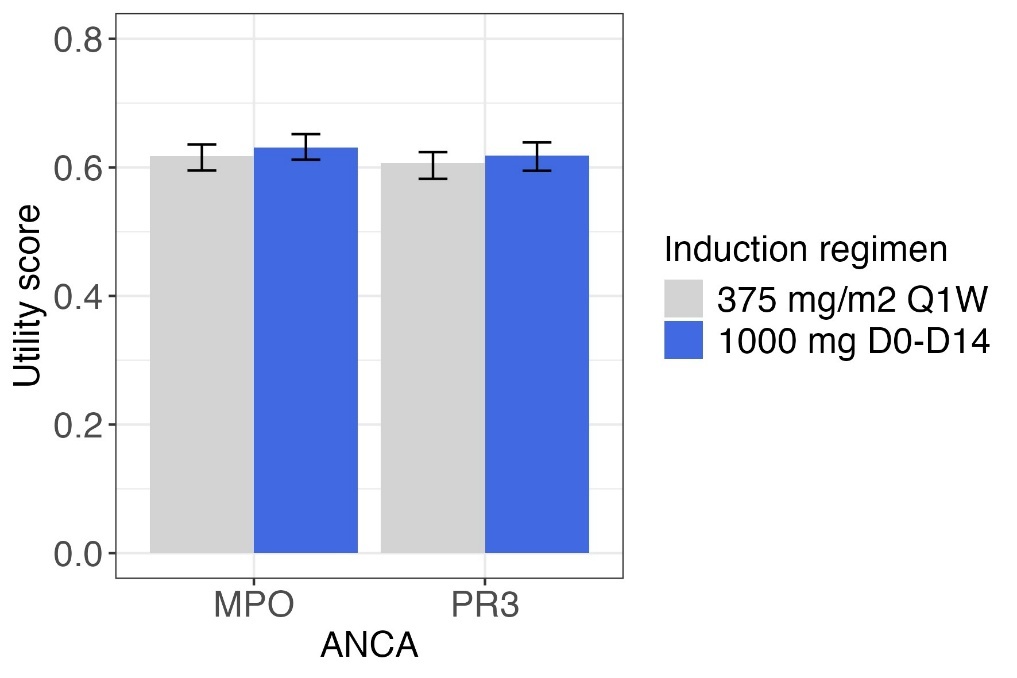


**Figure S10.** Clinical utility score for rituximab induction regimens with weighting allocation of 0.3/0.7 (efficacy/safety). Data are represented as median of 1000 simulations, the bars represent 90% prediction intervals.


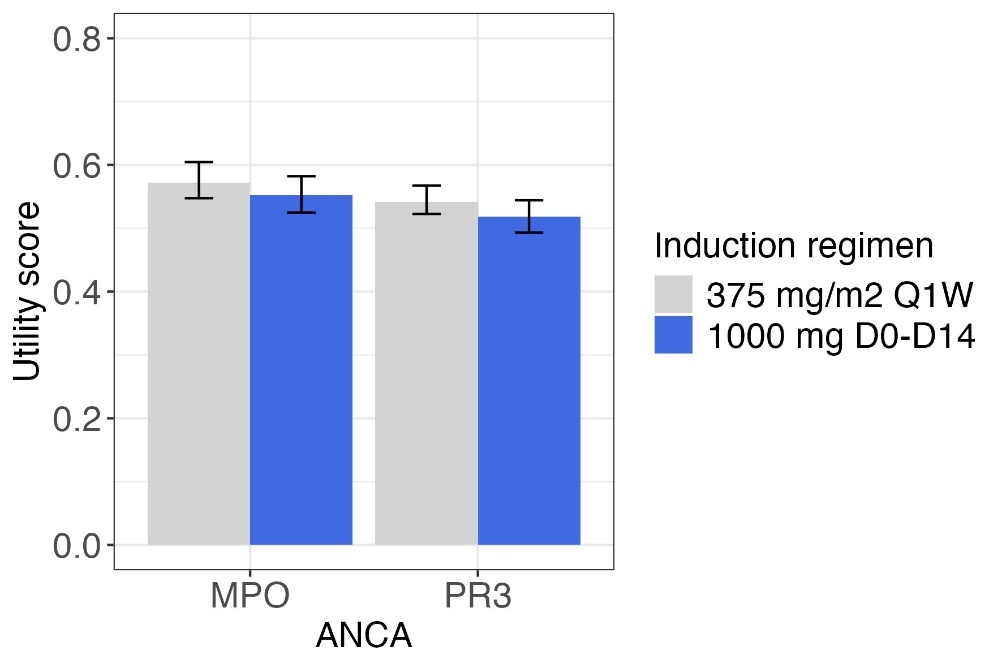


**Figure S11.** Clinical utility score for rituximab induction regimens with weighting allocation of 0.7/0.3 (efficacy/safety). Data are represented as median of 1000 simulations, the bars represent 90% prediction intervals.


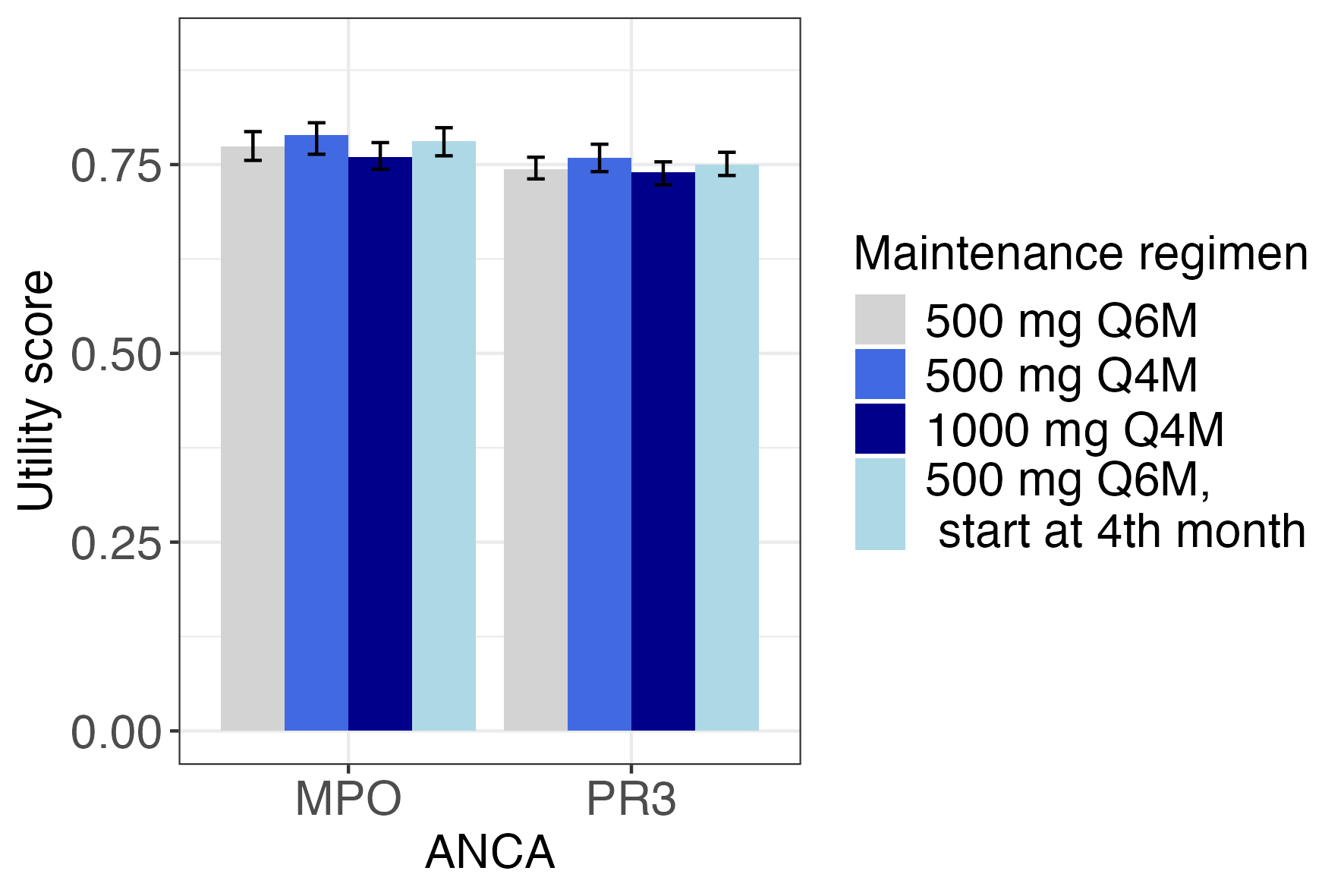


**Figure S12.** Clinical utility score for rituximab maintenance regimens with weighting allocation of 0.5/0.5 (efficacy/safety) considering patients with as serum gammaglobulins < 4 g/L. Data are represented as median of 1000 simulations and the bars represent 90% prediction intervals.


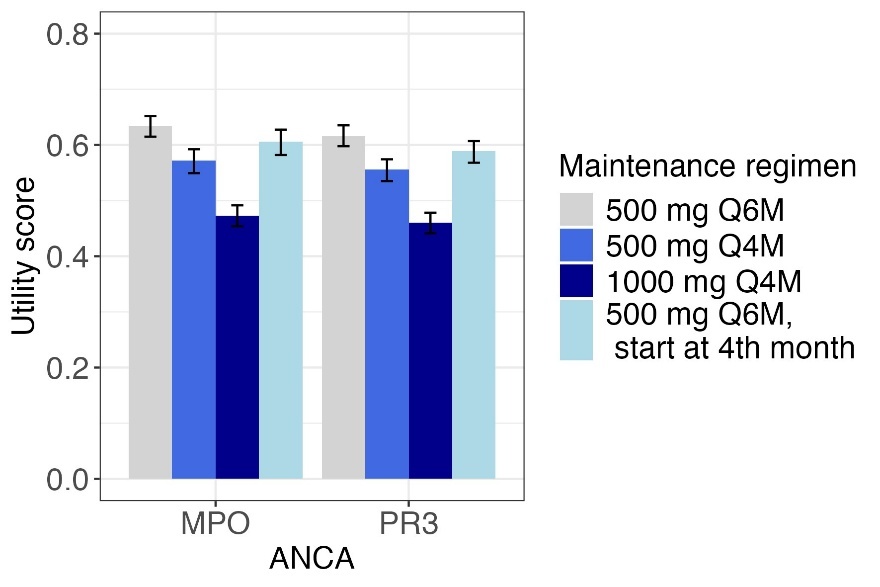


**Figure S13.** Clinical utility score for rituximab maintenance regimens with weighting allocation of 0.3/0.7 (efficacy/safety). Data are represented as median of 1000 simulations, the bars represent 90% prediction intervals.


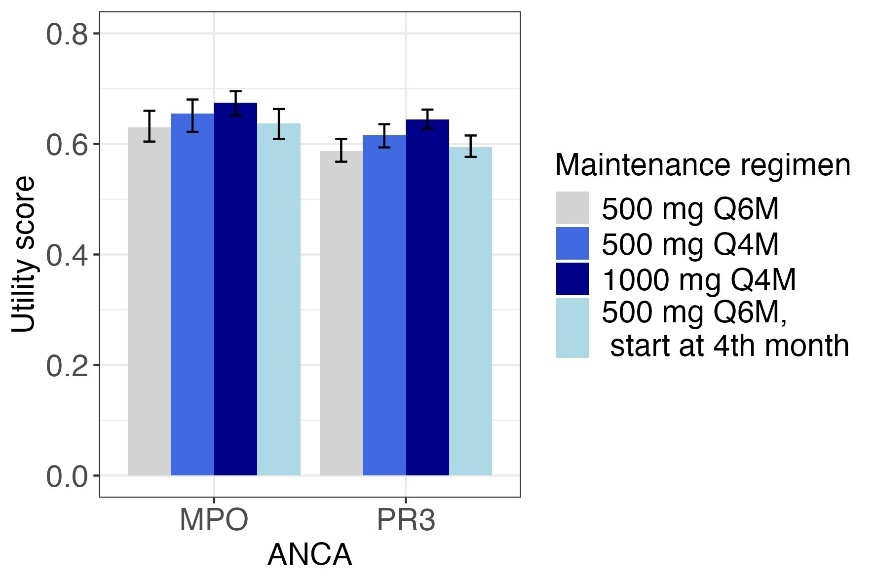


**Figure S14.** Clinical utility score for rituximab maintenance regimens with weighting allocation of 0.7/0.3 (efficacy/safety). Data are represented as median of 1000 simulations and the bars represent 90% prediction intervals.

**References**

1. Bergstrand M, Karlsson MO. Handling data below the limit of quantification in mixed effect models. AAPS J. 2009 Jun;11(2):371–80.

2. Bensalem A, Mulleman D, Paintaud G, Azzopardi N, Gouilleux-Gruart V, Cornec D, et al. Non-Linear Rituximab Pharmacokinetics and Complex Relationship between Rituximab Concentrations and Anti-Neutrophil Cytoplasmic Antibodies (ANCA) in ANCA-Associated Vasculitis: The RAVE Trial Revisited. Clin Pharmacokinet. 2020 Apr 1;59(4):519–30.

3. Ternant D, Monjanel H, Venel Y, Prunier-Aesch C, Arbion F, Colombat P, et al. Nonlinear pharmacokinetics of rituximab in non-Hodgkin lymphomas: A pilot study. Br J Clin Pharmacol. 2019 Sep 1;85(9):2002–10.

4. Rozman S, Grabnar I, Novaković S, Mrhar A, Jezeršek Novaković B. Population pharmacokinetics of rituximab in patients with diffuse large B-cell lymphoma and association with clinical outcome. Br J Clin Pharmacol. 2017 Aug 1;83(8):1782–90.

5. Petitcollin A, Bensalem A, Verdier MC, Tron C, Lemaitre F, Paintaud G, et al. Modelling of the Time-Varying Pharmacokinetics of Therapeutic Monoclonal Antibodies: A Literature Review. Clin Pharmacokinet. 2020 Jan 1;59(1):37–49.

6. Friberg LE, Henningsson A, Maas H, Nguyen L, Karlsson MO. Model of Chemotherapy-Induced Myelosuppression With Parameter Consistency Across Drugs. J Clin Oncol. 2002 Dec 15;20(24):4713–21.

7. Lejeune J, Raoult V, Dubrasquet M, Chauvin R, Mallebranche C, Pellier I, et al. Prediction of the Clinical Course of Immune Thrombocytopenia in Children by Platelet Kinetics. HemaSphere. 2023 Nov 27;7(11):E960.

8. Ayral G, Si Abdallah JF, Magnard C, Chauvin J. A novel method based on unbiased correlations tests for covariate selection in nonlinear mixed effects models: The COSSAC approach. CPT Pharmacometrics Syst Pharmacol. 2021 Apr 1;10(4):318–29.
